# Dynamics of Potential Distribution and Cultivation Areas of *Plantago asiatica* L. Under Climate Change: A Case Study of the Uppers of Dadu River—Minjiang River Basin

**DOI:** 10.1002/ece3.72172

**Published:** 2025-09-25

**Authors:** Yi Huang, Guanghua Zhao, Yang Yang, Jian Yang, Wu Zhi Jia Ba, Jia Lin Li

**Affiliations:** ^1^ Sichuan Provincial Forest and Grassland Key Laboratory of Alpine Grassland Conservation and Utilization of Tibetan Plateau, College of Grassland Resources Southwest Minzu University Chengdu China; ^2^ School of Life Sciences South China Normal University Guangzhou China; ^3^ Key Laboratory of Biodiversity and Environment on the Qinghai‐Tibetan Plateau, Ministry of Education, School of Ecology and Environment Tibet University Lhasa China; ^4^ Ecological Security and Protection Key Laboratory of Sichuan Province Mianyang, Normal University Mianyang Sichuan China

**Keywords:** climate change, ensemble modeling and production dynamics model, *Plantago asiatica* L., potential cultivation zone, potential distribution area

## Abstract

Global warming has induced significant shifts in spatiotemporal environmental patterns of plants. *P. asiatica* , highly prized for its edible and medicinal value, is widely harvested and utilized by residents in the upper reaches of the Dadu River and Minjiang River. This study employed ensemble models to simulate the potential distribution of *P. asiatica* in this region, predicting the impacts of future climate change on its distribution and niche. Additionally, a production dynamics model integrating the synergistic effects of ecological suitability and nutritional components of *P. asiatica* was developed to delineate its current and future potential cultivation zones. The results revealed the following: (1) Currently, both suitable habitats and cultivation zones of *P. asiatica* are primarily distributed in low‐altitude river valley areas within the upper reaches of the Minjiang River and Dadu River. (2) In future periods, high‐quality suitable habitats and cultivation zones of *P. asiatica* will increase to varying degrees, with its niche exhibiting a trend of migration toward higher‐altitude regions. (3) Under the SSP5‐8.5 climate emission scenario, the areas of suitable habitats and cultivation zones of *P. asiatica* will experience the greatest expansion, accompanied by the largest amplitude of niche migration. This study will facilitate the formulation of suitability‐based management strategies for *P. asiatica* in the upper reaches of the Dadu River and Minjiang River, and provide a scientific reference for the sustainable utilization of mountain plant resources under climate change.

## Graphical abstract

Climate change is reshaping the distribution of *Plantago asiatica* in the upper Dadu‐Minjiang River basin. Using ensemble and production dynamics models, this study predicts significant northward expansion of suitable habitats and cultivation zones under future climate scenarios, providing insights for sustainable resource management.

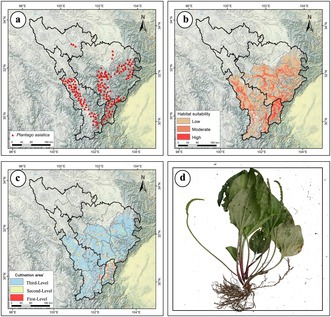

## 1 Introduction

Human utilization of plant resources under climate change has long attracted widespread attention, drawing interest from researchers in multiple disciplines, including geography, ecology, and archaeology (Yang, Gao, et al. 2024; Li et al. 2025; Wang, Zhuo, et al. 2025; Yang, Zhang, et al. 2022; Yang et al. 2025; Tian et al. 2025). As a special category of plant resources, herbivorous plant resources are closely linked to human development (Yang, Zhang, et al. 2022; Teixidor‐Toneu et al. 2018; Wan et al. 2024; Huang, Yang, Zhao, and Yang 2025). Factors influencing plant growth, development, and distribution have been extensively studied for decades and have become a hot topic in scientific research (Wang, Li, et al. 2025; Ramirez‐Barahona et al. 2025; Zhu et al. 2025; Zhao et al. 2025). The growth and development of herbivorous plant resources are affected by multiple factors, including climate, geomorphology, hydrology, and soil type (Yang et al. 2025; Brooks et al. 2006; Huang et al. 2022; Huang, Yang, Zhao, Shama, et al. 2025). Their nutritional components form the basis for their use as edible plants, and the richness of various nutrients and good flavor are prerequisites for their selection by local people (Feng et al. 2023; Huang, Yang, Zhao, Shama, et al. 2025). However, extreme climate change can adversely affect the metabolism, growth, and productivity of edible plants (Patni et al. 2022). Current studies have shown that among numerous influencing factors, nonclimatic factors only dominate short‐term biological changes in plants, whereas climate change is the main factor affecting their growth, development, and suitable distribution (Brooks et al. 2006; Liu et al. 2024). Climate change will cause significant changes and migration in the habitats of herbivorous plants and may also lead to changes in the content and quality of various nutrients in these plants (Feng et al. 2023; Huang, Yang, Zhao, Shama, et al. 2025).

The rapid development of species distribution models has enhanced our understanding of species niches and expanded our insights into their geographic distributions (Thuiller et al. 2009; Hao et al. 2019; Huang, Zhao, Yang, Yang, et al. 2025). Currently, research on the impact of climate on species distribution has been applied in various fields, including the spatiotemporal distribution of paleobiota, potential distribution of energy plants, conservation of endangered species, habitats of medicinal plants, and spatial distribution of crops (Huang et al. 2023, 2022; Liu et al. 2024; Lan et al. 2023; Huang, Yang, Zhao, Shama, et al. 2025; Liu et al. 2025). The ensemble modeling approach can significantly reduce prediction uncertainty when forecasting suitable habitats for species (Thuiller et al. 2009). For example, Lv et al. (2025) found that ensemble models yielded more stable predictions when assessing the climate suitability of *Prunus pseudocerasus* Lindl. in China. Huang, Yang, Zhao, Shama, et al. (2025) delineated cultivation zones of *Pteridium aquilinum var. latiusculum* in the eastern Qinghai‐Tibet Plateau using ensemble models, demonstrating that ensemble models achieved higher accuracy than single models. As an integrated platform, Biomod2 significantly improves the precision of species distribution predictions by fitting and comparing different models and evaluating the uncertainties (Bellard et al. 2012).

*Plantago asiatica* L. (Plantaginaceae) is a biennial or perennial herb distributed in temperate and tropical regions of the world. Native to Eurasia, it has been introduced to most regions of the world (van der Aart et al. 1992). It primarily grows in saline‐alkali soils, Gobi deserts, coastal tidal flats, wetlands, slopes, and grasslands below 4500 m elevation (Liu 2024; Yuan et al. 2024). This species is included in the *Pharmacopeia of the People's Republic of China* and is a common traditional Chinese medicinal herb. Modern pharmacological studies have revealed its immunomodulatory, anti‐inflammatory, and antioxidant properties (Wen et al. 2023). Additionally, it is a well‐known traditional wild vegetable and was listed as a health food by the Ministry of Health of China in 2002. Owing to topographic constraints and poor transportation, traditional livelihoods remain important in the daily lives of local residents in the upper reaches of the Dadu River and Minjiang River (Huang, Yang, Zhao, and Yang 2025; Huang, Yang, Zhao, Shama, et al. 2025). Field surveys indicate that the utilization of *P. asiatica* by residents in this region mainly involves two aspects: food and medicine (Text S1 and Figure S1). Each year, from April to May, local residents collect the tender aboveground parts of *P. asiatica* for consumption as a wild vegetable; it is also used as a tea to promote diuresis, relieve stranguria, clear heat, and detoxify (Zhang 2018) (Text S1). Relevant studies have shown that some wild edible plants in this region are significantly affected by climate change (Liu et al. 2022, 2025; Huang, Zhao, Yang, Yang, et al. 2025). Existing research on *P. asiatica* has mainly focused on its chemical components and pharmacological effects. However, as a major utilization region for *P. asiatica* , the mechanisms underlying its adaptation to climate change in the upper reaches of the Dadu River and Minjiang River remain unclear (Gao et al. 2024, 2025; Liu, Ma, et al. 2025; Qian et al. 2025).

This study, set in the upper reaches of the Dadu River and Minjiang River, focuses on *P. asiatica* and conducts the following work: (1) predict changes in *P. asiatica* 's suitable habitats under different climate scenarios in the upper Dadu River and Minjiang River basin; (2) analyze trends in its ecological niche dynamics; and (3) develop a suitability‐productivity model to delineate potential cultivation zones. These findings provide a theoretical basis for the sustainable utilization of this species in the region.

## 2 Materials and Methods

### 2.1 Sample Collection and Species Distribution Records

**FIGURE 1:**
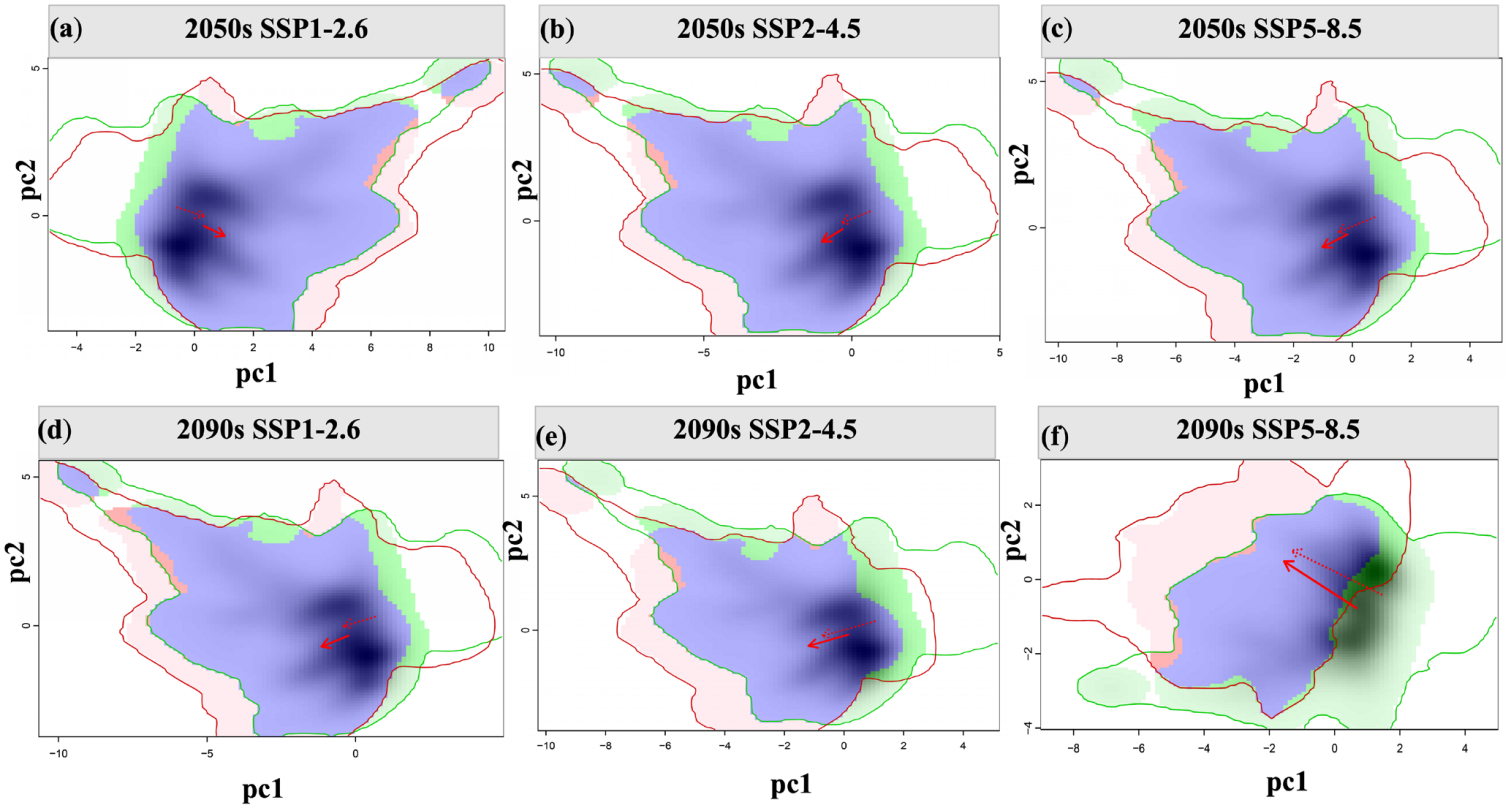
(a) Distribution records of *P. asiatica* in the upper Dadu River and Minjiang River basins; (b) current potential distribution area; (c) current cultivation zones; and (d) edible parts of *P. asiatica* .

Five field surveys were conducted in the upper Dadu River and Minjiang River basins between July 2022 and May 2024 to collect species distribution data, yielding 237 occurrence points for *P. asiatica* . To avoid model overfitting due to clustered points, only one point per 1 × 1 km grid was retained, resulting in 191 valid samples (Figure 1a) (the elevation, latitude, and longitude of each distribution point are shown in Table S1) (Zizka et al. 2019).

### 2.2 Selection and Processing of Environmental Variables

The study incorporated 41 environmental variables, including 19 bioclimatic factors, 16 soil factors, 3 topographic factors, 1 Human Footprint factor, 1 land use factor, and 1 NDVI factor. Both modern and future climate data were downloaded from the WorldClim database (http://worldclim.org/data/index.html, download time: August 2023, Original resolution 1 km^2^). Three scenarios (SSP126, SSP245, and SSP585) from the future climate system scenario models were applied, representing low, medium, and high greenhouse gas emission scenarios, respectively. Soil and topographic factor data were sourced from the Harmonized World Soil Database (HWSD) of the Food and Agriculture Organization of the United Nations (http://www.fao.org/faostat/en/#data, download time: August 2023, Original resolution 1 km^2^). Human Footprint data were obtained from the 2009 Human Footprint dataset, provided by the NASA Socioeconomic Data and Applications Center (SEDAC), which utilizes eight variables: built environments, population density, power infrastructure, croplands, pastures, roads, railways, and navigable waterways (original resolution 1 km^2^). The Normalized Difference Vegetation Index (NDVI) is the difference between near‐infrared and red reflectance values provided by the Land Processes Distributed Active Archive Center (LPDAAC) at the U.S. Geological Survey (USGS) Earth Resources Observation and Science (EROS) Center (http://LPDAAC.usgs.gov, download time: August 2023, Original resolution 250 m). Land use data were derived from the Resource and Environment Data Cloud Platform of the Chinese Academy of Sciences (http://www.resdc.cn/Default.aspx, download time: August 2023, Original resolution 1 km^2^). The spatial resolution of all factors was set to 1 km^2^ to ensure consistency in the environmental data.

To standardize the spatial resolution of all environmental variables to 1 km^2^, we used ArcGIS Pro software to resample data with an original resolution other than 1 km^2^ (e.g., the NDVI). Data with an original resolution of 1 km^2^ (e.g., climate, soil, topography, human footprint, and land use) were directly used or subjected to necessary unit conversions. Ultimately, the spatial resolution of all environmental factors was uniformly set to 1 km.

To avoid overfitting in model predictions caused by collinearity among environmental variables, the R language was used for Variance Inflation Factor (VIF) variable screening, principal component analysis (PCA) verification, and Spearman correlation tests. These steps aimed to improve the accuracy of the ecological niche model by reducing the model complexity (Zhao et al. 2021). The process consisted of four main steps: (1) Preliminary correlation screening: To mitigate the impact of multicollinearity on model accuracy, Spearman rank correlation analysis was first performed on all candidate environmental variables. Variables with a correlation coefficient threshold of |*r*| < 0.7 were retained for further analysis. (2) Handling highly correlated variables: For variable pairs with a Spearman correlation coefficient |*r*| ≥ 0.7, the variable with greater ecological significance or relevance to the research objective was retained, whereas its redundant counterpart was excluded. (3) Multicollinearity test: After the initial Spearman screening, the Variance Inflation Factor (VIF) was calculated for the remaining variables. Variables with a VIF ≥ 5, which indicate severe multicollinearity, were removed. (4) PCA verification: PCA was conducted on the variables retained after the previous two steps. The PCA results were used to visualize and validate the independence and data structure of the screened variables, ensuring that they effectively represented the main environmental variations while minimizing redundancy. This process yielded 16 variables (Table S2).

### 2.3 Construction and Accuracy Evaluation of Ensemble Models

In this study, the biomod2 package was used to construct ensemble models, which require species presence and pseudo‐absence data. Using the method provided by biomod2 to generate absence points from background data, the “random” method was employed to randomly generate 1290 pseudo‐absence points for modeling (Figure S2). The biomod2_tuning function was used to optimize each algorithm automatically. The biomod2_tuning‐optimized model parameters were applied to randomly select 75% of the sample data as training data, with the remaining 25% used for model validation. The weights of the presence and pseudo‐absence data were set to be equal, with 10 repetitions. Using the weighted average method, only models with a TSS ≥ 0.7 were retained to construct the ensemble model. For the filtered models, the weighted average method (em.algo = ‘weighted.mean’) was adopted, where the weights were determined by the TSS values of each model, ultimately constructing a single ensemble model.

This study used the built‐in bm_FindOptimStat function to convert continuous values into binary values via a threshold to obtain the optimal score for the given evaluation. When using the prediction function BIOMOD_EnsembleForecasting, we set the parameter metric.binary = “TSS” to obtain binary maps. From the model results, a 0/1 threshold (cutoff = 0.355) was derived: areas below the threshold were classified as unsuitable habitats, while areas above the threshold were divided into three grades: low, moderate, and high suitability. The Distribution Change Between Binary SDMs tool in the SDM Tools plugin of ArcGIS was used to calculate changes in the niche area across different periods.

Species distribution models often overestimate or underestimate species distributions, resulting in false positives and negatives. False positives refer to cases in which the model predicts non‐distribution areas as potential distribution areas, whereas false negatives are the opposite. Therefore, many scholars have used various evaluation metrics to assess model accuracy (Huang, Yang, Zhao, and Yang 2025; Liu et al. 2024). ROC values, TSS values, and Kappa statistics are commonly used accuracy evaluation metrics for species distribution modeling. Thus, this study also selected these three metrics to validate the accuracy of the simulations of each model.

### 2.4 Ecological Niche Dynamics

Under the current climate conditions, background point selection areas were defined using *P. asiatica* occurrence points with a 1‐degree buffer. For future scenarios, background areas were based on ensemble‐modeled suitable habitat. Niche overlap rates between current and future niches were calculated using the *ecospat* package, with niche parameter *D* (ranging from 0 to 1, where 0 = no overlap, 1 = complete overlap) visualized to analyze climate change impacts (Liu et al. 2024).

### 2.5 Establishing Relationships Between Cultivation Productivity and Environmental Suitability

In this study, food science methods were used to randomly determine the nutritional components (general nutritional components, bioactive substances, and amino acid compositions) of *P. asiatica* from 36 distribution points. Referring to previous research methods (Huang, Yang, Zhao, and Yang 2025; Huang, Yang, Zhao, Shama, et al. 2025), a cultivation productivity evaluation model integrating the synergistic effects of ecological suitability and nutritional components of *P. asiatica* was constructed, as described.

(1) P=S+N

To evaluate the relationship between the cultivation productivity and environmental suitability of *P. asiatica* , the ecological suitability value (S, Suitability) was derived from the presence probability values output by the species distribution model. Suitability data for each cultivation site were extracted via spatial interpolation, and weights were assigned to them. For the nutritional components (N, Nutrients), indicator weights were determined using the entropy weight method. Details on the types of nutritional components of *P. asiatica* , the assigned weight proportions, and the rationale are provided in Text S2. After standardization of each indicator (Table S3), weighted summation was performed using the following formula:

(2) $$N=\sum_{i=1}^{4}w_{i}\cdot X_{i}^{\mathrm{norm}}$$

For model validation, the ggtrendline package in R was used to fit the quantitative relationship between cultivation productivity and ecological suitability using seven types of nonlinear regression models (Table S4) (Sun et al. 2021). The optimal model was selected based on the Akaike Information Criterion (AIC) (ΔAIC < 2). Finally, the distribution of potential cultivation zones of *P. asiatica* under current and future climatic conditions was predicted using the optimal model (Huang, Zhao, Yang, Yang, et al. 2025).

## 3 Results

### 3.1 Multi‐Model Prediction Outcomes and Ensemble Model Accuracy Validation

Overall, model simulations for *P. asiatica* showed suitable habitats primarily concentrated in the eastern, southern, and central regions of the upper Dadu River and Minjiang River basin. Although all models agreed on the general trend, the predictive results varied significantly across the models (Figure S3). The biomod_tuning function optimized the model parameters, with parameter validation performed using ROC, Kappa, and TSS metrics in each iteration. The ensemble model (Ensemble) achieved exceptional accuracy: mean TSS = 0.943, ROC = 0.994, and KAPPA = 0.911, outperforming the individual models (Figure S4). These results confirm the superior fitting and predictive performance of the ensemble model.

### 3.2 Current Potential Distribution of *P. asiatica* in the Upper Dadu River and Minjiang River Basin

The potential distribution of *P. asiatica* in the study area is shown in Figure 1b, with a total suitable habitat area of 7.52 × 10^4^ km^2^ (Table S5). High‐suitability zones (0.47 × 10^4^ km^2^, 6.25% of the total) occurred as patchy distributions in Tianquan and Baoxing Counties, with linear distributions across Luhuo, Daofu, Markam, Heishui, Xiaojin, and Jinchuan Counties (Figure 1b). Medium‐suitability zones (1.96 × 10^4^ km^2^, 26.06% of the total) formed patchy and linear peripheries around high‐suitability areas (Figure 1b). It can be concluded that under current climatic conditions, *P. asiatica* is mainly distributed in low‐altitude areas in the upper reaches of the Dadu River and Minjiang River.

### 3.3 Future Potential Distribution of *P. asiatica* in the Upper Dadu River and Minjiang River Basin

**FIGURE 2:**
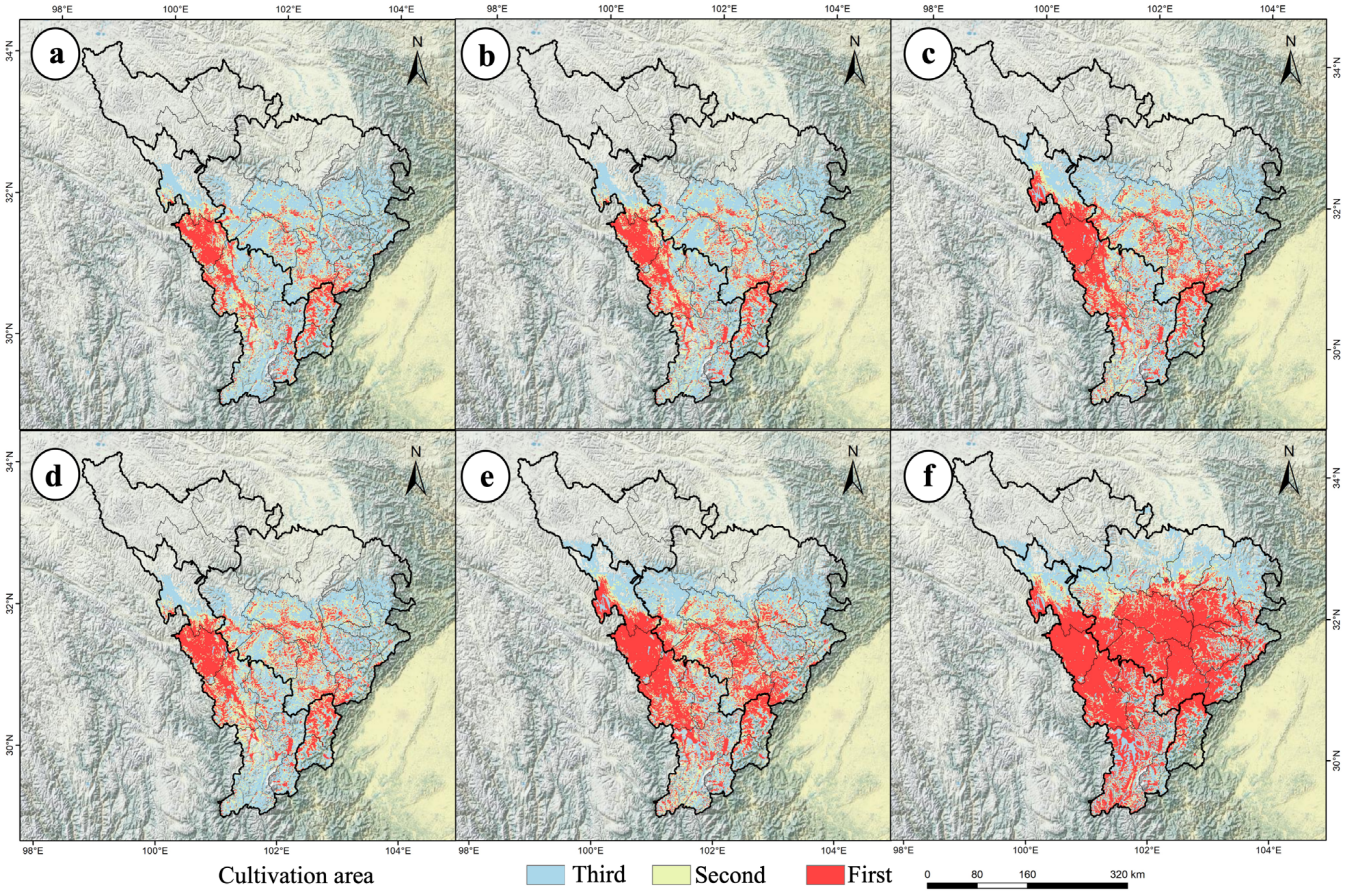
Changes in the potential geographic distribution of *P. asiatica* under future climate scenarios in the upper Dadu River and Minjiang River basin. (a) 2050s, SSP1‐2.6; (b) 2050s, SSP2‐4.5; (c) 2050s, SSP5‐8.5; (d) 2090s, SSP1‐2.6; (e) 2090s, SSP2‐4.5; (f) 2090s, SSP5‐8.5.

The ensemble model projected *P. asiatica* 's potential distributions under SSP1‐2.6, SSP2‐4.5, and SSP5‐8.5 scenarios for 2050 and 2090 (Figure 2), with area changes summarized in Table S5. The total suitable area increased the most under SSP5‐8.5 in the 2090s (73.25% increase, 5.50 × 10^4^ km^2^), with the smallest increase under SSP1‐2.6 in the 2050s (21.28% increase, 1.60 × 10^4^ km^2^). High‐suitability area exhibited explosive growth under SSP5‐8.5 (1565.96% increase, 7.36 × 10^4^ km^2^ in the 2050s), versus the smallest increase under SSP1‐2.6 (429.79% increase, 2.02 × 10^4^ km^2^ in the 2050s). Medium‐suitability area peaked under SSP1‐2.6 (71.43% increase, 1.40 × 10^4^ km^2^ in the 2090s) but decreased significantly under SSP5‐8.5 (16.84% reduction, 0.33 × 10^4^ km^2^ in the 2090s). Low‐suitability areas declined under all scenarios, with the largest reduction under SSP5‐8.5 (43.03% decrease, 2.19 × 10^4^ km^2^ in the 2090s). It can be concluded that under climate change, the suitable habitats of *P. asiatica* in the upper reaches of the Dadu River and Minjiang River show a polarized trend, characterized by an extreme increase in highly suitable areas and a systematic decrease in poorly suitable areas.

**FIGURE 3:**
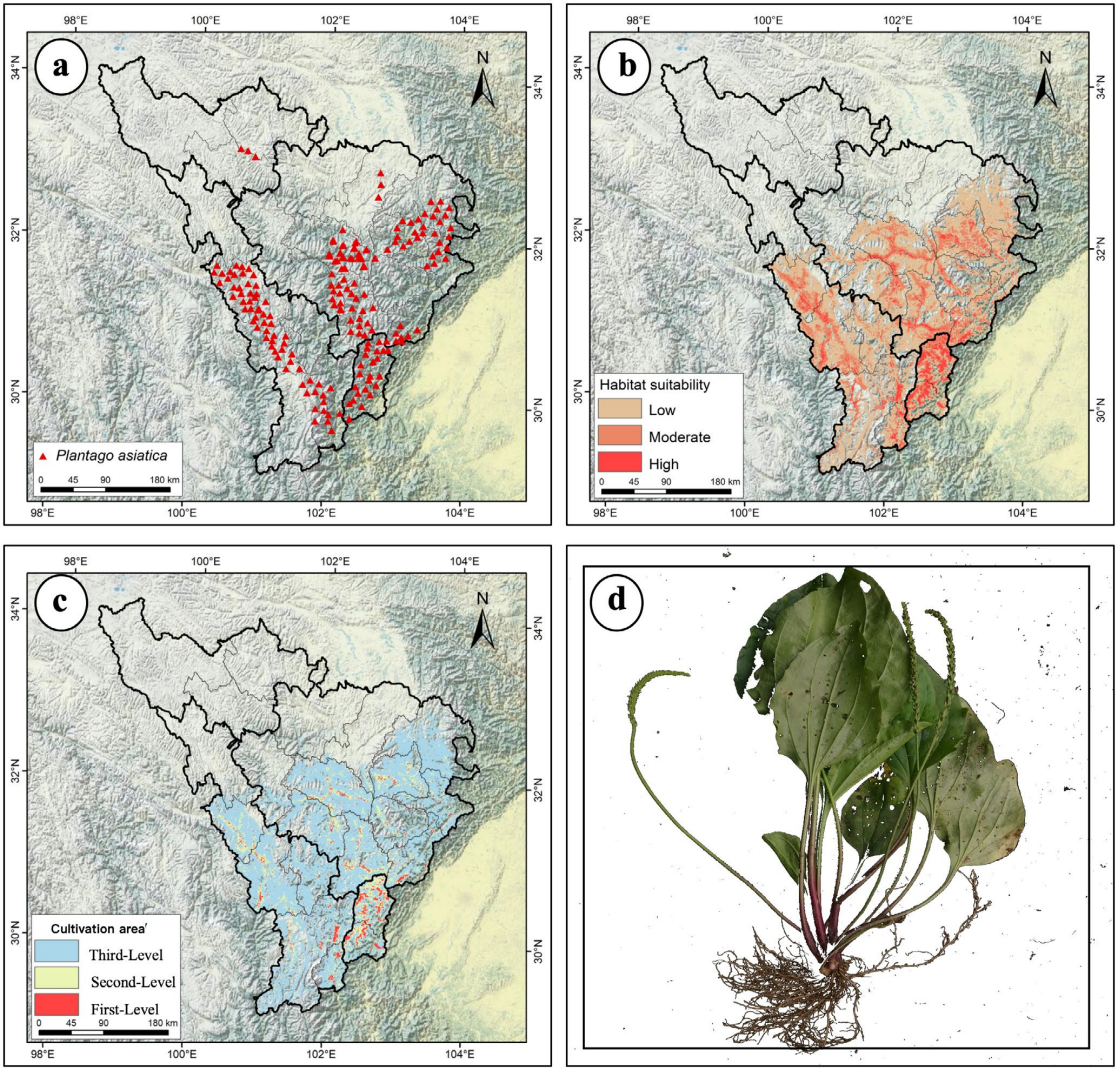
Basinsiotemporal shifts in the potential geographic distribution of *P. asiatica* under climate change in the upper Dadu River and Minjiang River basin. (a) 2050s, SSP1‐2.6; (b) 2050s, SSP2‐4.5; (c) 2050s, SSP5‐8.5; (d) 2090s, SSP1‐2.6; (e) 2090s, SSP2‐4.5; (f) 2090s, SSP5‐8.5.

These trends indicate a bipolar pattern of “extreme high‐suitability expansion and systematic low‐suitability contraction” under climate change (Figure 2). Spatial overlay analysis in ArcGIS revealed a continuous range expansion under all scenarios (Figure 3). By the 2050s, SSP5‐8.5 drove the largest expansion (41.94%, 3.15 × 10^4^ km^2^), while SSP1‐2.6 showed the smallest (27.81%, 2.09 × 10^4^ km^2^). By the 2090s, SSP5‐8.5 increased expansion to 75.93% (5.71 × 10^4^ km^2^), compared to 31.24% under SSP1‐2.6 (2.35 × 10^4^ km^2^) (Table S6, Figure 3). The distribution range of *P. asiatica* expanded significantly with increasing emission intensity.

### 3.4 Analysis of Future Ecological Niche Changes

**FIGURE 4:**
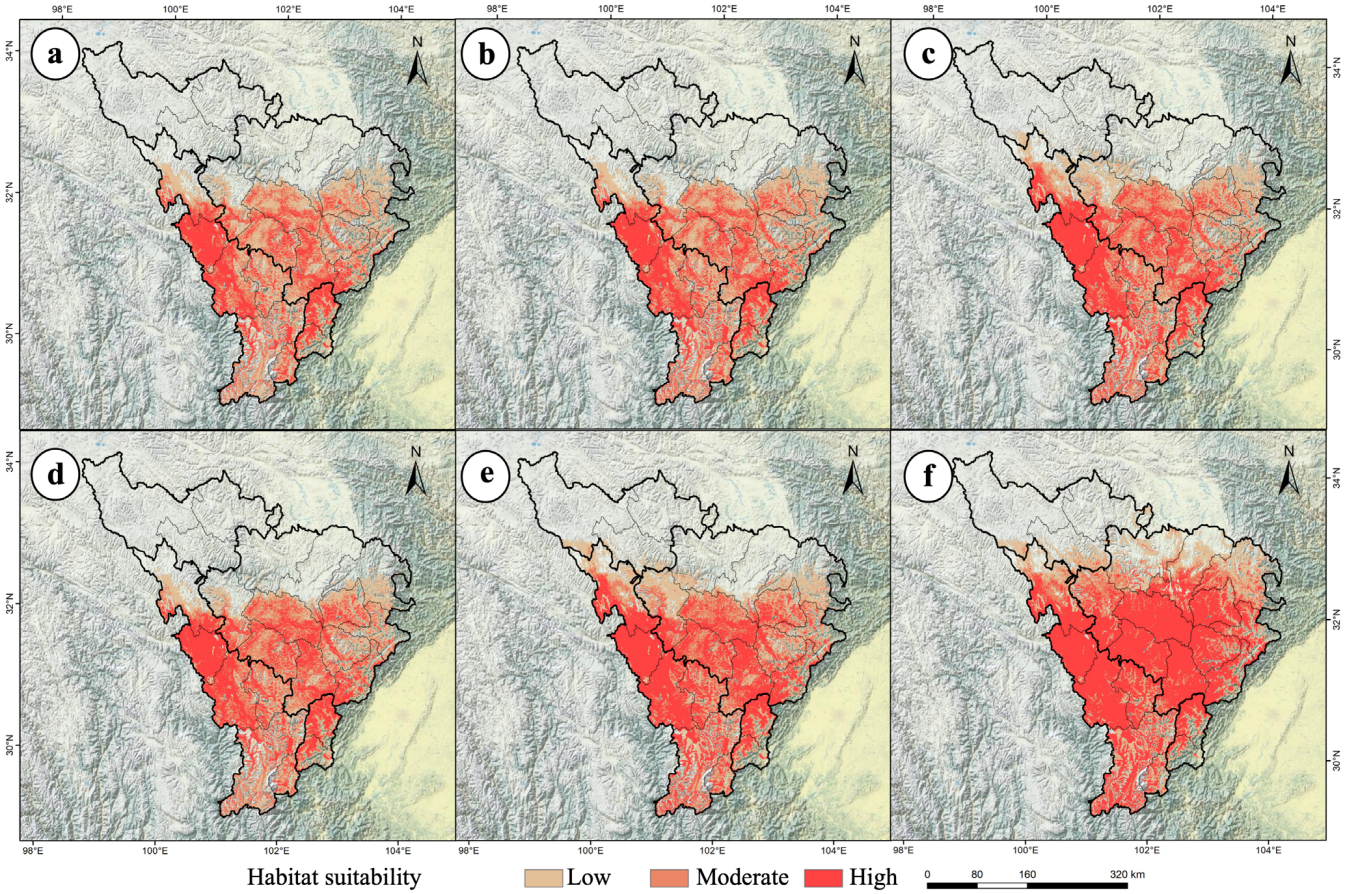
Future niche changes of *P. asiatica* under different climatic scenarios. PC1 and PC2 represent the first two axes of the principal component analysis (PCA). Green and red shading indicates species occurrence density in the current and future scenarios, respectively; blue denotes overlap. Solid and dashed contour lines represent 100% and 50% of the available environmental space, respectively. The red arrows illustrate shifts in the climatic niche centroid (solid line) and background range centroid (dashed line). (a) 2050s, SSP1‐2.6; (b) 2050s, SSP2‐4.5; (c) 2050s, SSP5‐8.5; (d) 2090s, SSP1‐2.6; (e) 2090s, SSP2‐4.5; (f) 2090s, SSP5‐8.5.

Niche overlap of *P. asiatica* in the upper Dadu River and Minjiang River basin is shown in Figure 4. The climatic niche shifts under future climate scenarios mirrored background climate trends, with larger migration distances and lower niche equivalence under SSP5‐8.5 than under SSP1‐2.6 and SSP2‐4.5. By the 2090s under SSP5‐8.5, *P. asiatica* 's niche will be significantly decoupled from the current period. The increasing magnitude of climate change has driven pronounced niche shifts, as evidenced by declining niche overlap. Principal component analysis (PCA) revealed that the first two components explained 68.69%–72.10% of environmental variance (PC1:51.53%–53.31%; PC2:17.16%–18.79%), with temperature seasonality (coefficient of variation), annual temperature range, and soil gravel content as the primary drivers of niche changes. The future climatic niche centroid is projected to shift toward a higher annual temperature range and temperature seasonality. Overall, *P. asiatica* 's niche shifted northwestward toward higher latitudes across all three emission scenarios from the baseline to the 2050s and 2090s, with the largest centroid migration and high‐suitability area expansion under high‐emission scenarios.

### 3.5 Dynamics of Potential Cultivation Zones for *P. asiatica* Across Periods

According to the Akaike Information Criterion (AIC), the linear model depicted in Figure S3c was identified as the optimal model among the seven tested models. Figure S5 shows a significant positive correlation between the habitat suitability and productivity of *P. asiatica* .

**FIGURE 5:**
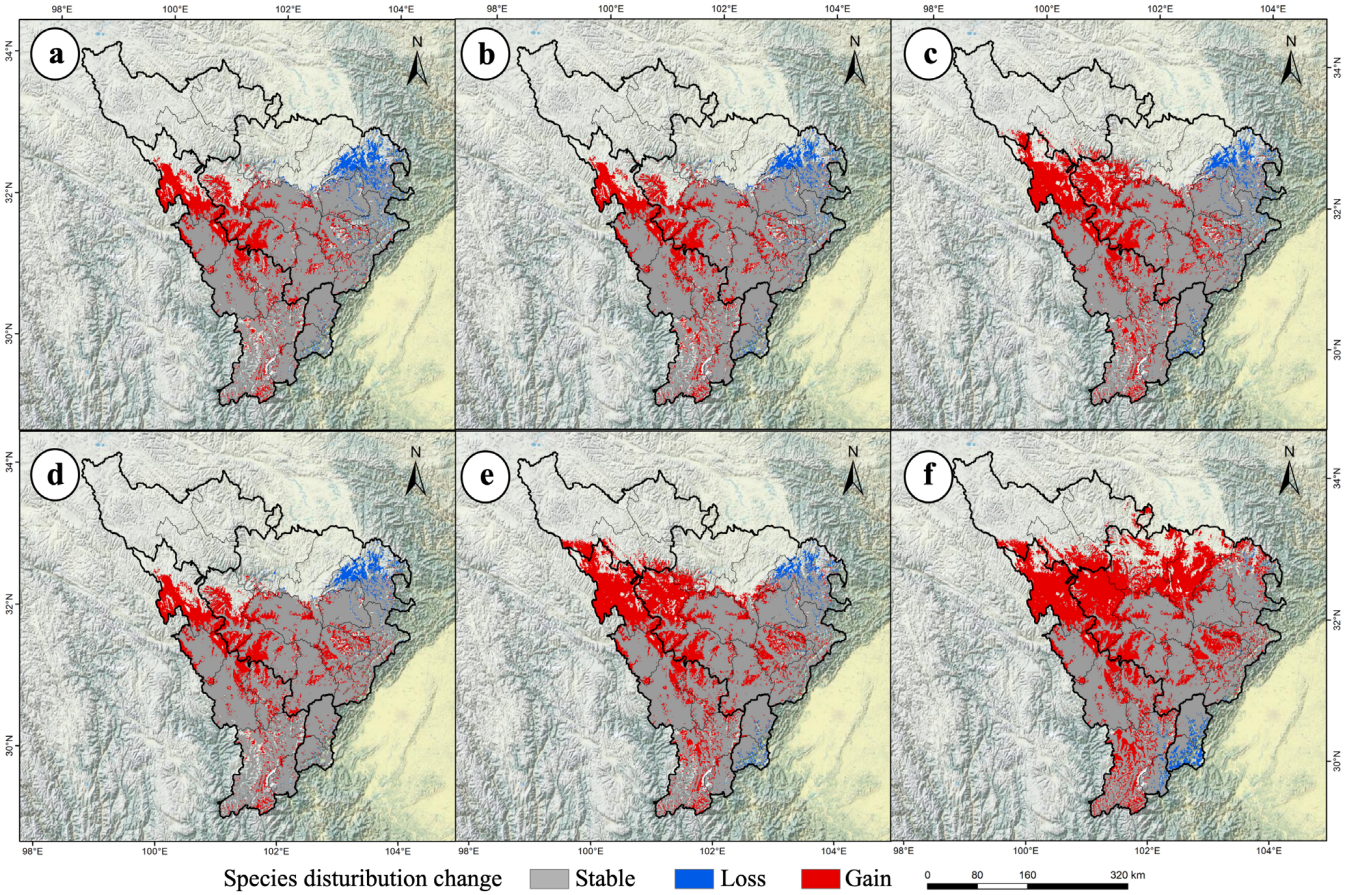
Distribution of three‐tier cultivation zones for *P. asiatica* in the upper Dadu River and Minjiang River basin under future climate scenarios. (a) 2050s, SSP1 to 2.6; (b) 2050s, SSP2 to 4.5; (c) 2050s, SSP5 to 8.5; (d) 2090s, SSP1 to 2.6; (e) 2090s, SSP2 to 4.5; (f) 2090s, SSP5 to 8.5.

Productivity was classified into three tiers (high, medium, and low) using the production dynamics model. High‐productivity areas were designated as first‐level cultivation zones, medium‐productivity as second‐level, and low‐productivity as third‐level cultivation zones. Under current climate conditions, the total cultivation area reached 6.80 × 10^4^ km^2^, including 0.23 × 10^4^ km^2^ (first‐level) and 0.89 × 10^4^ km^2^ (second‐level) zones (Table S7). The current cultivation zones are concentrated in the entire upper Minjiang River and low‐elevation areas of the upper Dadu River, with first‐level zones primarily in eastern Tianquan County and southern Baoxing County (Figure 1c). Quantitative analysis of cultivation zone dynamics under different Shared Socioeconomic Pathways (SSPs) revealed pronounced changes across all three tiers (Figure 5). Cultivation zone expansions intensified over time and emission intensity in both target periods (the 2050s and the 2090s). The most significant increases occurred under SSP5‐8.5: by the 2050s, the total cultivation area under SSP5‐8.5 increased to 9.79 × 10^4^ km^2^, with first‐level zones expanding to 2.82 × 10^4^ km^2^ and second‐level zones to 2.48 × 10^4^ km^2^ (Table S7). By the 2090s, the total cultivation area under SSP5‐8.5 surged to 12.58 × 10^4^ km^2^, with first‐level zones reaching 7.00 × 10^4^ km^2^ and second‐level zones reaching 1.95 × 10^4^ km^2^ (Table S7).

These trends indicate the northward migration of cultivation zones toward higher latitudes, driven by rising temperatures and altered precipitation patterns. In summary, all climate scenarios projected consistent expansion across all cultivation tiers, with explosive growth of first‐level zones under SSP5‐8.5. This suggests a substantial conversion of non‐cultivation areas to second/third‐level zones and the upgrading of lower‐tier zones to first‐level zones under extreme emissions.

## 4 Discussion

Ecological niche models utilize known species distribution data and environmental variables to infer the ecological requirements of species via algorithms, projecting the results onto the corresponding spatiotemporal domains to simulate the actual and potential distributions (Xie et al. 2023). In recent years, single‐species distribution models, such as discriminant analysis or support vector machines, have been criticized for their limitations, such as overfitting, high uncertainty, and reliance on specific data types (Zhao et al. 2021). Ensemble models address these issues by integrating the strengths of individual models, separating output results, uncertainties, and errors, and mapping dominant trends across models (Thuiller 2003). However, ensemble performance is not universally superior to single models, as it depends on factors such as species traits, model selection criteria, and environmental variable sampling errors (Hao et al. 2019), which must be considered during model construction. This study selected optimal single models based on prediction outcomes, evaluation metrics, and the actual distribution of *P. asiatica* to construct an ensemble model. The ensemble model achieved a mean TSS of 0.943, ROC of 0.994, and KAPPA of 0.911, outperforming the single models. This indicates that ensemble modeling leverages the complementarity among single models to reduce bias and uncertainty, yielding more accurate predictions. Additionally, *P. asiatica* 's suitable habitats in the study area align with distribution heatmaps from iPlant (https://www.iplant.cn), and field survey records fall within predicted suitable zones, further validating simulation accuracy.

In this study, temperature seasonality (coefficient of variation), annual temperature range, and soil gravel content were identified as the core environmental factors driving niche differentiation in *P. asiatica* (Table S2; Figure S6). This is highly consistent with the known biological characteristics of *P. asiatica* . Yuan et al. (2024) noted that, as a widely distributed temperate species, *P. asiatica* exhibits extreme sensitivity in its phenological rhythms and physiological metabolism to seasonal temperature fluctuations. Additionally, soil gravel content directly influences colonization ability by regulating water permeability and root development space. Against the backdrop of global warming, the upper reaches of the Dadu River and Minjiang River, located on the eastern edge of the Qinghai‐Tibet Plateau, are experiencing significant climatic anomalies (Chen et al. 2011; Guo et al. 2020). Gao (2018) confirmed that future temperature increases in this region will be pronounced, leading to an expanded annual temperature range and intensified seasonal fluctuations. Such changes have a profound impact on the distribution of herbaceous plants. Huang, Yang, Zhao, and Yang (2025); Huang, Yang, Zhao, Shama, et al. (2025) similarly identified the decisive role of these temperature factors in shaping the niches of alpine herbs in studies conducted on the eastern edge of the Qinghai‐Tibet Plateau, corroborating the vulnerability of plants in this region to drastic climate changes.

The strong response of *P. asiatica* to temperature factors is further reflected in its niche dynamics. With the increasing intensity of climate change, the degree of niche overlap between current and future climate scenarios decreases, indicating a significant shift in the niche. Chen and Liu (2019) investigated the effects of temperature and precipitation on the growth of *P. asiatica* , supporting the above conclusions. Furthermore, the critical role of soil factors has been confirmed in this study. Xiao et al. (2018) compared the photosynthetic physiological performance of *P. asiatica* in grassland, understory, and riparian habitats and found that riparian sandy soil habitats are the most suitable for its growth. This is consistent with the conclusion of this study that soil gravel content dominates local niche differentiation in this region. In summary, temperature changes and soil physical structure are core environmental variables that must be prioritized when managing and delineating future cultivation zones for *P. asiatica* .

Climate change may accelerate species extinction, reduce biodiversity, and destabilize regional ecosystems, although some species may evolve new physiological traits to adapt (Garcia et al. 2013; Carosi et al. 2020). Global climate and environmental change research has gained increasing scientific prominence in recent decades (Karypidou et al. 2020; Yang, Jiang, et al. 2024). Under three emission scenarios for 2050 and 2090, the potential geographic distribution and cultivation areas of *P. asiatica* generally expanded compared to the current conditions, with drastic increases in high‐suitability zones and first‐level cultivation areas. Thomas et al. (2004) noted that climate warming is a double‐edged sword for species; while some may benefit, others face risks. Clearly, *P. asiatica* in the upper Dadu River and Minjiang River basin benefits from warming, as evidenced by its distribution and growth trends.

The upper Dadu River and Minjiang River basin is located in Southwest China (Zhang 2018). Numerous studies in Southwest China have shown that plants tend to migrate northward during climate warming. For example, Yang, Jiang, et al. (2022) simulated the potential distribution of *Magnolia wilsonii* and found that the impacts of climate change became more pronounced with increasing greenhouse gas emissions, driving its migration to higher latitudes under sustained warming. *Fritillariae Cirrhosae Bulbus* is projected to experience severe habitat fragmentation and substantial northwestward and high‐latitude migration under climate change (Liu et al. 2022), whereas *Paeonia decomposita* exhibits overall habitat suitability shifts (Liu et al. 2025). The Shared Socioeconomic Pathway (SSP) scenarios proposed by the IPCC indicate that the scope of climate warming and temperature increases will become more evident in the future (Lee et al. 2023). Under the SSP5‐8.5 emission scenario, rising temperatures driven by higher emission concentrations may amplify suitable habitat changes for *P. asiatica* , potentially explaining the drastic expansion of high‐suitability zones and first‐level cultivation areas, as well as the largest niche migration under this scenario. The impacts of climate warming on species' potential geographic distributions are primarily characterized by shifts to higher latitudes or altitudes and changes in range expansion or contraction (Thuiller 2003). The northward and high‐latitude shift of *P. asiatica* 's potentially suitable habitats in this study aligns with this pattern.

The projected expansion of *P. asiatica* 's suitable habitats under climate change presents opportunities for sustainable resource use. Our suitability‐productivity model provides a scientific basis for the cultivation zoning. The current cultivation zones in the upper Minjiang River and Kangding, Daofu, and Danba Counties (Figure 1c) match the hydrothermal requirements of the species. Future warming will reshape cultivation patterns, with SSP5‐8.5 driving the explosive growth of first‐level zones in high‐altitude/latitude areas (Figure 5). Although this offers the potential for large‐scale cultivation, it also challenges resource management. As a valued medicinal and edible plant (Zhang 2018), *P. asiatica* could shift from wild harvesting to artificial cultivation in the current first‐level zones, reducing the pressure on wild resources. Adaptive management strategies, such as dynamic cultivation zoning and climate‐driven northward migration monitoring (Figure 5), are essential for maintaining high‐quality planting areas.

The upper reaches of the Dadu River—Minjiang River are a transition zone between forests and grasslands on the Qinghai‐Tibet Plateau (Huang, Yang, Zhao, Shama, et al. 2025; Zhang 2018). This study showed that the niche of *P. asiatica* tends to migrate from the current forest‐grassland transition zone to high‐elevation grasslands, with a much greater migration amplitude under high‐emission scenarios than under low‐emission scenarios. The stability of grassland ecosystems is much lower than that of forest and shrub ecosystems (Sun et al. 2021). The large‐scale exploitation and utilization of *P. asiatica* in grassland ecosystems will undoubtedly pose a huge challenge to the ecological stability and diversity of grasslands. Therefore, in future exploitation and utilization of *P. asiatica* in the upper reaches of the Dadu River—Minjiang River, the possibility of its migration to high‐elevation grassland areas under climate change needs to be considered.

This study provides a macro‐planning basis for the sustainable management of *P. asiatica* resources in alpine canyon regions under climate change scenarios, but several aspects still require further refinement. Future research can be expanded through multi‐dimensional explorations. At the level of fundamental mechanisms, it is necessary to deepen studies on the coupling mechanisms of multiple global change factors and to analyze the comprehensive impacts of their interactions on the population dynamics and adaptability of *P. asiatica* . Meanwhile, applications of molecular ecology and conservation genetics should be expanded; technologies such as genomics and transcriptomics can be used to clarify the genetic basis of key adaptive traits, population genetic structure, and gene flow patterns, thereby assessing the current status of genetic diversity and adaptive potential to future environmental changes. Intelligent monitoring and early warning technologies should be developed at the technical support level. A high‐precision real‐time monitoring system for resource dynamics and an early warning system for habitat degradation should be constructed by integrating drone remote sensing, near‐surface sensor networks, and deep learning. Management practice: Community‐participatory adaptive co‐management models must be explored. Combining traditional knowledge and livelihood needs, community‐based management that integrates climate‐smart agriculture, ecological conservation compensation, and sustainable harvesting certification should be designed in the future. However, cross‐scale habitat connectivity restoration should be strengthened. Considering the geomorphic characteristics of alpine canyons, near‐natural ecological corridor restoration schemes based on key habitat corridors and stepping‐stone patches should be researched and designed to alleviate habitat fragmentation and enhance the resilience of ecosystems. Ultimately, scenario analysis and policy evaluation tools must be integrated to develop a comprehensive platform that couples ecosystem service assessment, cost–benefit analysis, and multi‐agent modeling. This platform will simulate the dynamic responses of resource sustainability and ecosystem services under different climate change scenarios, management policy interventions, and socioeconomic development pathways, providing a quantitative basis and dynamic adjustment schemes for adaptive management decisions.

## 5 Conclusions

This study focuses on *P. asiatica* , a medicinal and edible plant, in the upper Dadu River and Minjiang River basin. Using ensemble modeling and multi‐source environmental data, we systematically analyzed the dynamics and driving mechanisms of *P. asiatica* 's suitable habitats under three current to future emission scenarios. A production dynamics model was developed to evaluate the spatial patterns of ecological suitability and nutritional components, delineating three cultivation zones for the species in the study area. Our results show that with increasing emission scenario severity, the areas of high‐suitability habitats and first‐level cultivation zones for *P. asiatica* will significantly expand in the upper Dadu River and Minjiang River basin. We recommend prioritizing the establishment of artificial planting bases in current first‐level cultivation zones to meet market demands through standardized cultivation and reduce reliance on wild resources. These findings provide a scientific basis for adaptive management strategies and future industrial planning for *P. asiatica* .

## Author Contributions

**Yi Huang:** conceptualization (equal), data curation (equal), formal analysis (equal), supervision (equal), writing – original draft (equal). **Yang Yang:** data curation (equal), supervision (equal). **Guanghua Zhao:** data curation (equal), investigation (equal), software (equal). **Jian Yang:** conceptualization (equal), supervision (equal), writing – review and editing (equal). **Wu Zhi Jia Ba:** data curation (equal), investigation (equal). **Jia Lin Li:** data curation (equal), investigation (equal).

## Conflicts of Interest

The authors declare no conflicts of interest.

## Supporting information


**Data S1:** ece372172‐sup‐0001‐supinfo.docx.

## Data Availability

All the required data are available as Supporting Information.
