# Supplementary material for: Dynamics of Potential Distribution and Cultivation Areas of Plantago asiatica L. Under Climate Change: A Case Study of the Uppers of Dadu River—Minjiang River Basin
Source: Ecol Evol. 2025 Sep 25;15(10):e72172. doi: 10.1002/ece3.72172 (PMC12463456; doi:10.1002/ece3.72172)
Supplement: Supplementary file 1 — Data S1: ece372172‐sup‐0001‐supinfo.docx. [file ECE3-15-e72172-s001.docx]

**Supplementary material for**

**Dynamics of Potential Distribution and Cultivation Areas of *Plantago asiatica* L. Under Climate Change: A Case Study of the Upper Dadu-Minjiang River Basin**

**TEXT S1. Survey on the Importance and Multi-Uses of *P. asiatica*.**

In this study, initial informants were randomly selected in the upper reaches of the Dadu River and Minjiang River. After interviewing these informants, subsequent informants were recommended by the previous ones for further interviews. Through this process, a total of 409 informants were included in the team’s survey to collect traditional knowledge on the utilization of wild edible plant resources (Figure S1).

The Relative Frequency of Citation (RFC) was used to investigate the utilization of *P. asiatica* in the upper reaches of the Dadu River and Minjiang River. When considering the use categories of *P. asiatica*, RFC refers to the proportion of respondents who mentioned a specific use of the species relative to the total number of respondents, calculated as:

RFC=FC/N （1）

where FC is the number of respondents reporting a specific use, and N is the total number of respondents.

The diuretic effect of *P. asiatica* is widely recognized. Its seedlings are blanched and served cold, and it can also be brewed into tea to clear heat and detoxify. The survey results showed that *P. asiatica* has three main uses in the upper reaches of the Dadu River and Minjiang River, with RFC values ranked as follows: medicinal use (RFC = 0.99) > edible use (RFC = 0.82) > tea use (RFC = 0.45). This indicates that edible and medicinal uses are the primary utilization aspects in this region.

**TEXT S2: Types of Nutritional Components in *P. asiatica*, Weight Ratios Assigned, and Rationale**
 In this study, routine nutritional components, bioactive substances, and amino acid profiles of *P. asiatica* were measured. After several discussions by experts from the Sichuan Provincial Key Laboratory of Ecology and the Sichuan Provincial University Engineering Center for Disaster Prevention and Mitigation, it was determined that weights should be assigned to component categories based on their nutritional functions, health values, and environmental sensitivities.

A total weight of 100% was allocated as follows:

- **Routine nutrients and bioactive components (75% total)**:
  - **Protein (12%)**: As a core indicator of growth and quality, its synthesis is significantly regulated by nitrogen use efficiency.
  - **Dietary fiber (10%)**: Reflects soil and moisture conditions through cell wall component regulation and supports intestinal health.
  - **Fat (7%)**, **crude fiber (5%)**, and **ash (1%)**: Allocated based on energy density, stress resistance, and mineral content, respectively.
  - **Starch (15%)**: A core component of carbohydrate metabolism, its synthesis is regulated by light and temperature via key enzymes such as ADP-glucose pyrophosphorylase.
  - **Total flavonoids (12%)**: Enhance antioxidant capacity via UV-B-induced phenylpropanoid metabolism.
  - **Total saponins (8%)** and **tannins (5%)**: Indicate immune potential and stress response, respectively.
- **Amino acid components (25% total)**:
  - **Essential amino acids (12%)**: Each essential amino acid accounts for 1.5%, reflecting their dependency on nitrogen metabolism efficiency.
  - **Conditionally essential amino acids (5%)**:
    - Arginine (2%): Involved in salt stress response.
    - Cysteine (1.5%): Reflects sulfur metabolism levels.
    - Tyrosine (1.5%): Indicates secondary metabolism regulatory capacity.
  - **Glutamic acid (4%)**: Serves as a nitrogen metabolism hub in salt stress regulation.
  - **Other non-essential amino acids (4%)**:
    - Proline (2%): Responds to drought via osmotic adjustment.
    - Remaining amino acids: 0.5% each.


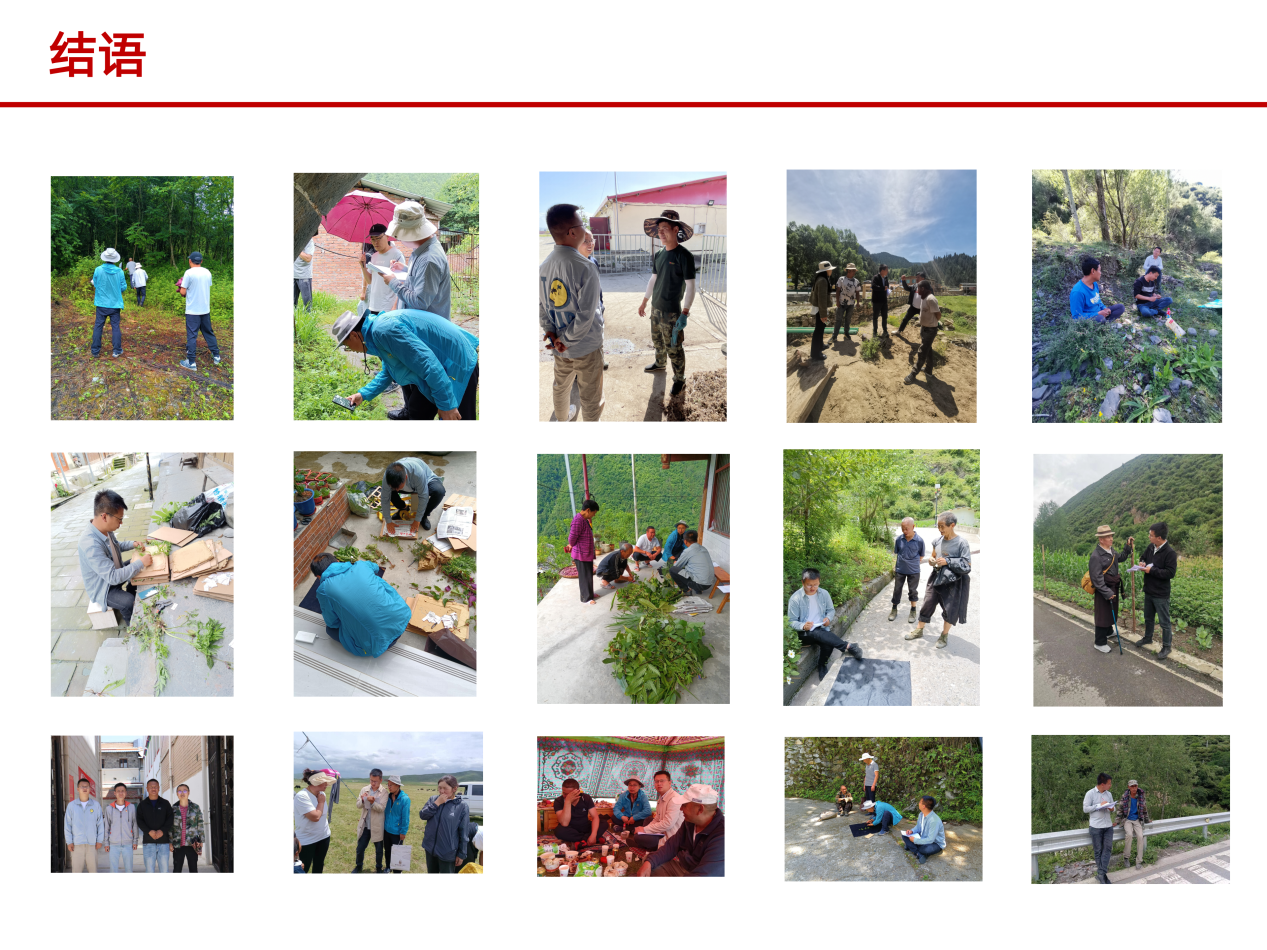


**FIGURE S1** Field Investigation of the Upper Reaches of Dadu River and Minjiang River.


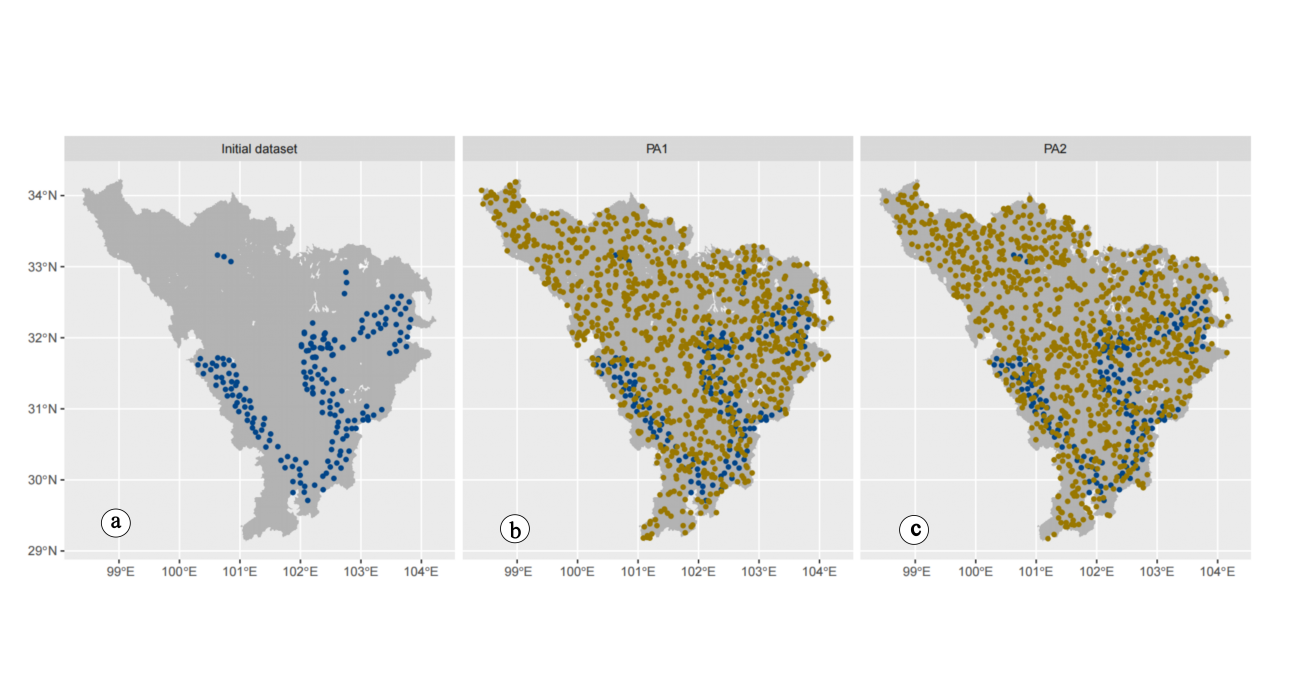


**FIGURE S2** The actual occurrence points and 1,290 pseudo-absence points data of  *P. asiatica*. (a) Actual occurrence points; (b) First repetition result of pseudo-absence points; (c) Second repetition result of pseudo-absence points. Note: Blue represents actual occurrence points data, and brown represents pseudo-absence points data.


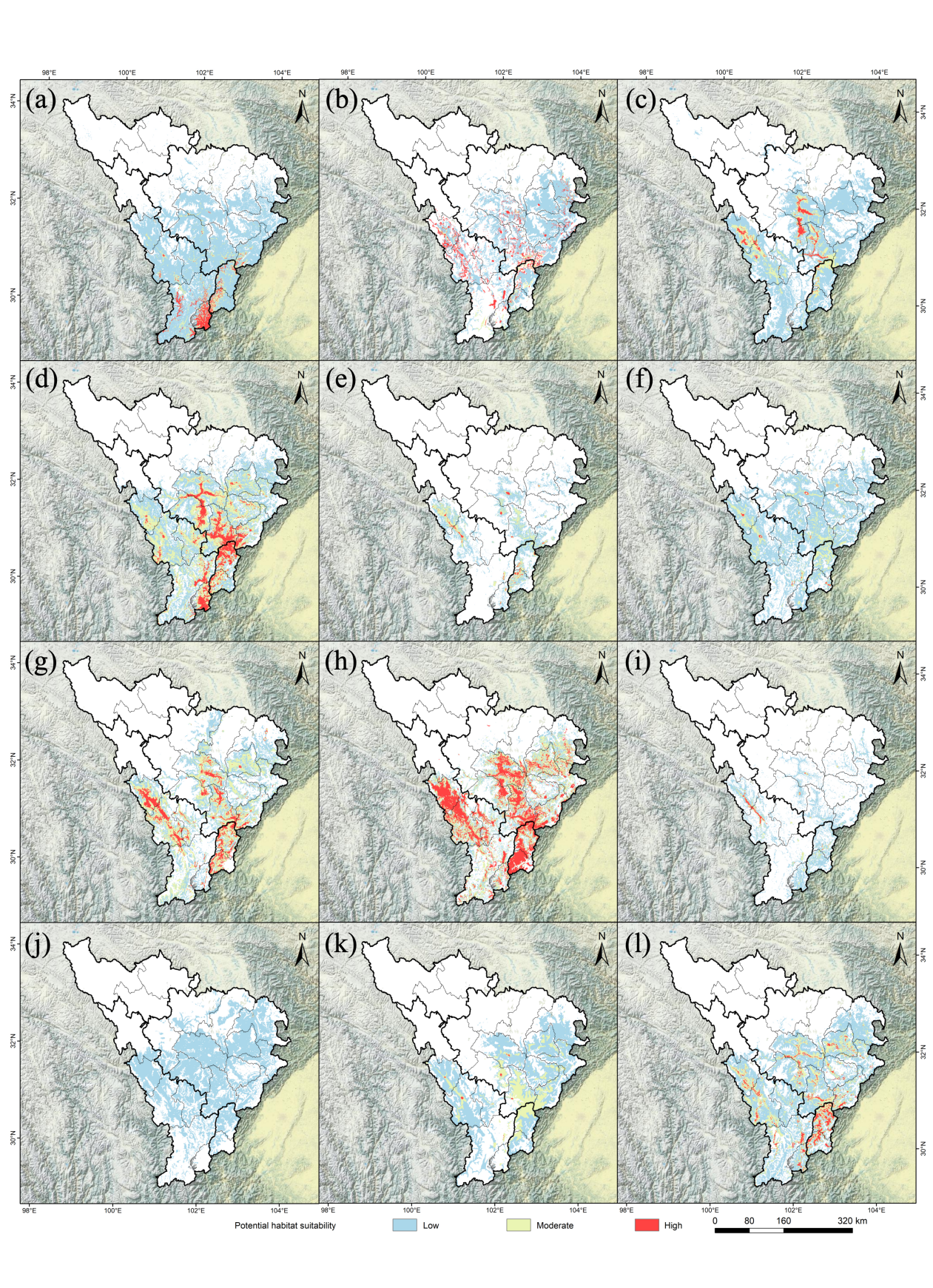


**FIGURE S3** Multi-model prediction of the potential distribution of *P. asiatica* in the upper Dadu-Minjiang River basin. (a) Artificial Neural Network (ANN) model, (b) Generalized Typical Analysis (GTA) model, (c) Flexible Discriminant Analysis (FDA) model, (d) Generalized Additive Model (GAM), (e) Gradient Boosting Machine (GBM) model, (f) Generalized Linear Model (GLM), (g) Multivariate Adaptive Regression Splines (MARS) model, (h) MaxEnt model, (i) Random Forest (RF) model, (j) Semi-Parametric Regression (SER) model, (k) XGBOOST model, (l) Ensemble model.

**
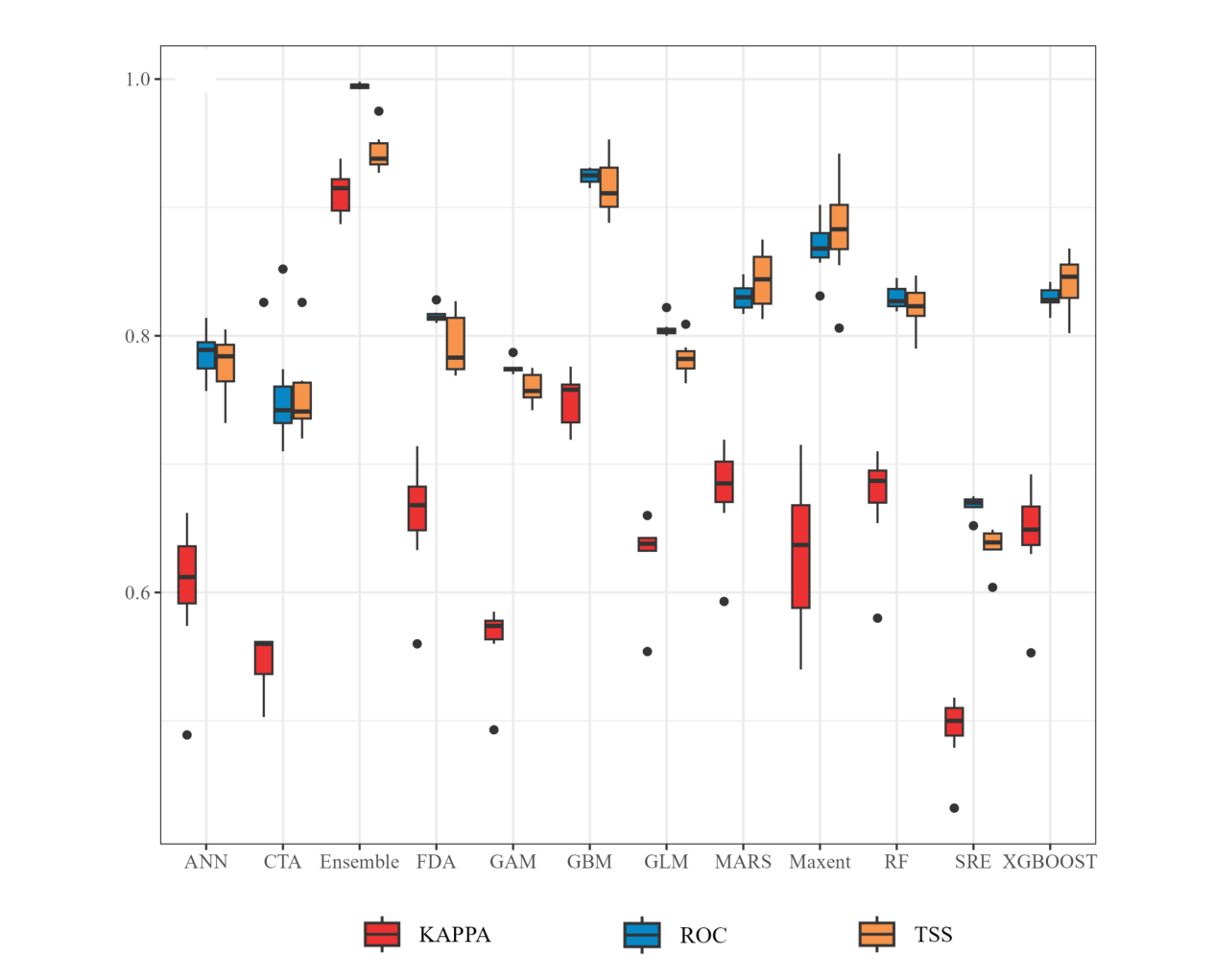
**

**FIGURE S4** Evaluation of model accuracy based on three evaluation indicators


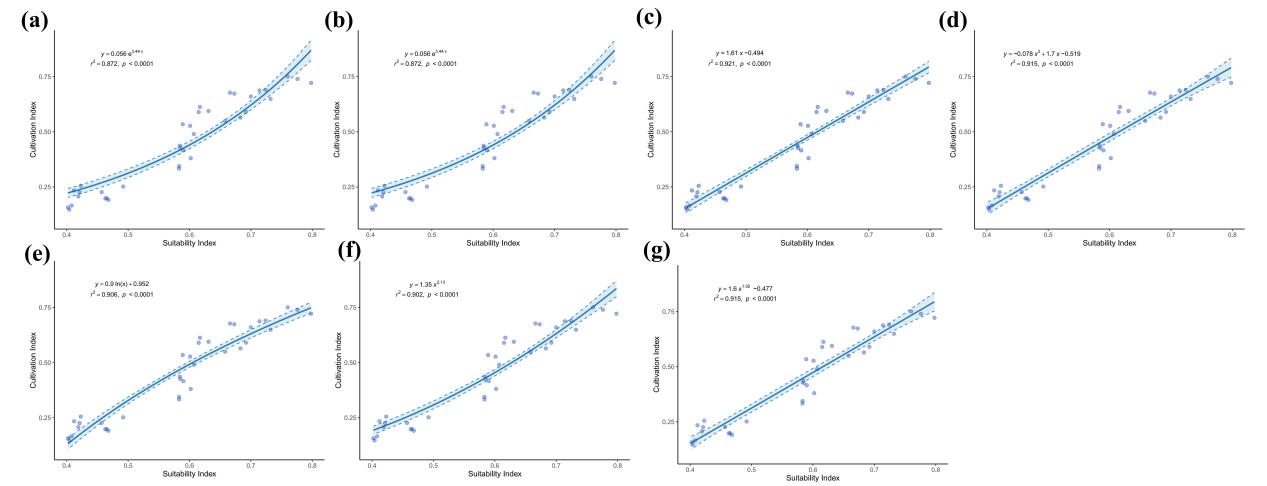


**FIGURE S5** Relationship between suitability and productivity of *P. asiatica*


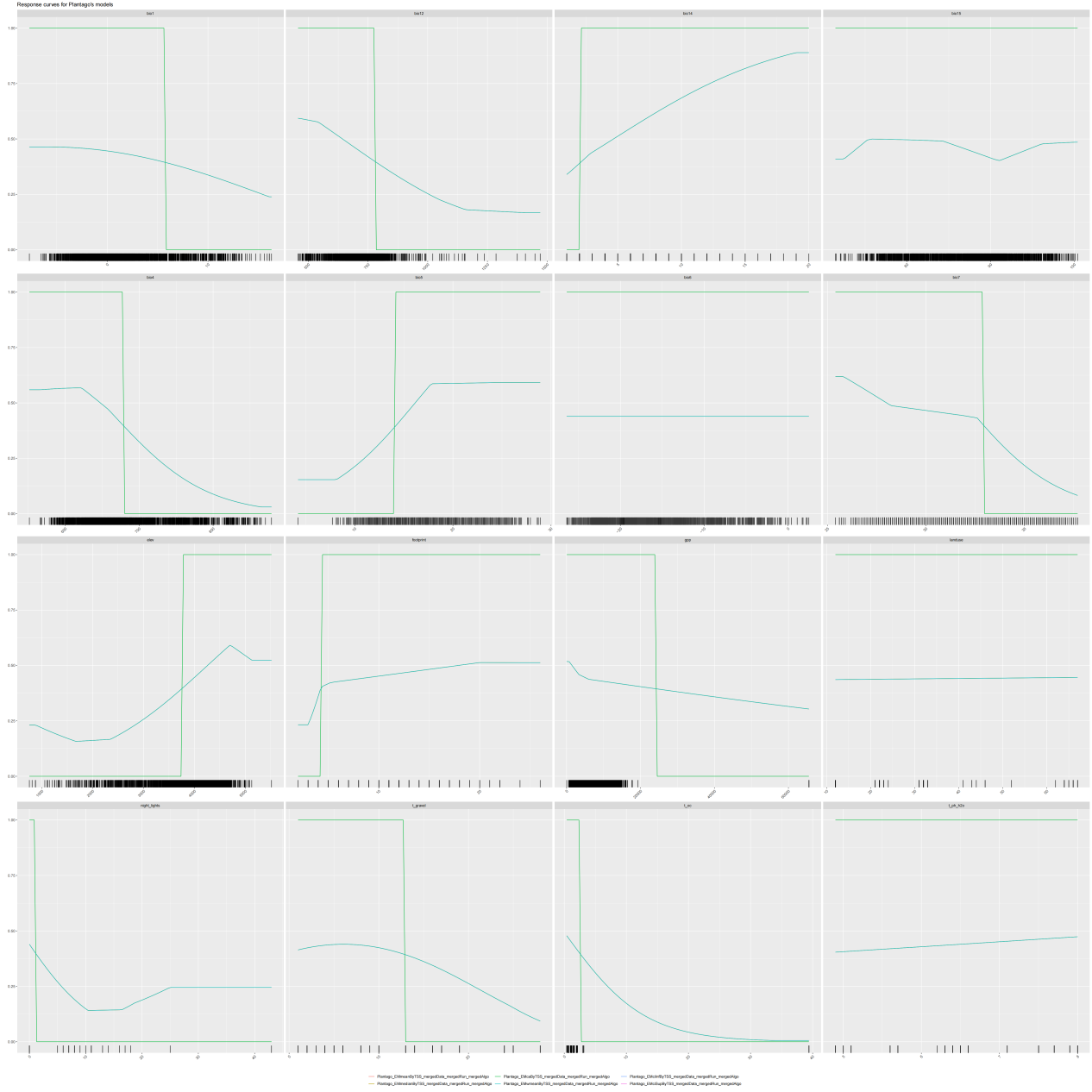


**FIGURE S6** Response curves of the probability of occurrence of *P. asiatica* to modeling environmental variables

**TABLE S1.** Latitude, longitude, and altitude of distribution sites included in the modeling of *P. asiatica*

| **Number** | **longitude** | **latitude** | **Number** | **longitude** | **latitude** | **Number** | **longitude** | **latitude** | **Number** | **longitude** | **latitude** |
| --- | --- | --- | --- | --- | --- | --- | --- | --- | --- | --- | --- |
| **1** | 100.628 | 33.163 | **49** | 102.064 | 32.077 | **97** | 102.204 | 32.008 | **145** | 100.857 | 31.281 |
| **2** | 100.736 | 33.141 | **50** | 102.221 | 32.013 | **98** | 102.356 | 31.849 | **146** | 100.878 | 31.192 |
| **3** | 100.851 | 33.071 | **51** | 102.374 | 31.976 | **99** | 102.174 | 31.912 | **147** | 100.697 | 31.439 |
| **4** | 102.753 | 32.921 | **52** | 102.458 | 31.911 | **100** | 102.209 | 31.859 | **148** | 100.722 | 31.372 |
| **5** | 102.763 | 32.776 | **53** | 102.449 | 31.861 | **101** | 102.241 | 31.729 | **149** | 100.761 | 31.273 |
| **6** | 102.728 | 32.621 | **54** | 102.539 | 31.762 | **102** | 102.017 | 31.877 | **150** | 100.791 | 31.181 |
| **7** | 103.522 | 32.578 | **55** | 102.329 | 31.857 | **103** | 102.101 | 31.816 | **151** | 100.611 | 31.445 |
| **8** | 103.665 | 32.584 | **56** | 102.174 | 31.921 | **104** | 102.787 | 30.831 | **152** | 100.605 | 31.331 |
| **9** | 103.617 | 32.484 | **57** | 102.225 | 31.864 | **105** | 102.895 | 30.837 | **153** | 101.091 | 31.285 |
| **10** | 103.798 | 32.504 | **58** | 102.016 | 31.901 | **106** | 102.919 | 30.725 | **154** | 101.163 | 31.111 |
| **11** | 103.739 | 32.413 | **59** | 102.134 | 31.821 | **107** | 102.851 | 30.723 | **155** | 101.183 | 31.016 |
| **12** | 103.431 | 32.428 | **60** | 102.257 | 31.724 | **108** | 102.758 | 30.717 | **156** | 101.233 | 30.868 |
| **13** | 103.558 | 32.398 | **61** | 102.398 | 31.551 | **109** | 102.766 | 30.622 | **157** | 101.406 | 30.865 |
| **14** | 103.658 | 32.331 | **62** | 102.549 | 31.414 | **110** | 102.703 | 30.576 | **158** | 101.388 | 30.778 |
| **15** | 103.823 | 32.256 | **63** | 102.684 | 31.251 | **111** | 102.626 | 30.811 | **159** | 101.359 | 30.701 |
| **16** | 103.798 | 32.147 | **64** | 102.359 | 31.419 | **112** | 102.612 | 30.746 | **160** | 101.507 | 30.646 |
| **17** | 103.767 | 32.012 | **65** | 102.432 | 31.362 | **113** | 102.593 | 30.668 | **161** | 101.489 | 30.555 |
| **18** | 103.753 | 31.876 | **66** | 102.534 | 31.211 | **114** | 102.527 | 30.536 | **162** | 101.627 | 30.468 |
| **19** | 103.584 | 32.186 | **67** | 102.608 | 31.058 | **115** | 102.514 | 30.427 | **163** | 100.997 | 31.191 |
| **20** | 103.657 | 32.074 | **68** | 102.676 | 30.973 | **116** | 102.804 | 30.405 | **164** | 101.065 | 31.126 |
| **21** | 103.642 | 31.961 | **69** | 102.376 | 31.239 | **117** | 102.754 | 30.287 | **165** | 101.081 | 31.026 |
| **22** | 103.561 | 31.905 | **70** | 102.488 | 31.111 | **118** | 102.665 | 30.403 | **166** | 101.123 | 30.924 |
| **23** | 103.585 | 31.811 | **71** | 102.491 | 31.019 | **119** | 102.651 | 30.348 | **167** | 101.122 | 30.838 |
| **24** | 103.476 | 31.782 | **72** | 102.493 | 30.929 | **120** | 102.601 | 30.238 | **168** | 101.209 | 30.804 |
| **25** | 103.333 | 32.359 | **73** | 102.571 | 30.909 | **121** | 102.671 | 30.167 | **169** | 101.207 | 30.732 |
| **26** | 103.225 | 32.317 | **74** | 102.359 | 31.089 | **122** | 102.553 | 30.021 | **170** | 101.258 | 30.671 |
| **27** | 103.098 | 32.337 | **75** | 102.363 | 30.949 | **123** | 102.376 | 29.862 | **171** | 101.304 | 30.603 |
| **28** | 103.018 | 32.246 | **76** | 102.292 | 31.481 | **124** | 102.454 | 30.262 | **172** | 101.429 | 30.462 |
| **29** | 103.412 | 32.265 | **77** | 102.239 | 31.593 | **125** | 102.494 | 30.182 | **173** | 100.981 | 31.177 |
| **30** | 103.403 | 32.188 | **78** | 102.203 | 31.723 | **126** | 102.428 | 30.094 | **174** | 100.953 | 31.086 |
| **31** | 103.288 | 32.195 | **79** | 102.058 | 31.656 | **127** | 102.371 | 30.052 | **175** | 100.923 | 31.039 |
| **32** | 103.327 | 32.133 | **80** | 102.166 | 31.519 | **128** | 100.345 | 31.706 | **176** | 100.981 | 30.961 |
| **33** | 103.208 | 32.081 | **81** | 102.186 | 31.421 | **129** | 100.306 | 31.619 | **177** | 102.091 | 30.241 |
| **34** | 103.116 | 32.021 | **82** | 102.201 | 31.298 | **130** | 100.427 | 31.611 | **178** | 102.235 | 29.926 |
| **35** | 103.026 | 32.137 | **83** | 102.203 | 31.226 | **131** | 100.545 | 31.641 | **179** | 101.918 | 30.286 |
| **36** | 102.995 | 32.074 | **84** | 102.057 | 31.516 | **132** | 100.633 | 31.717 | **180** | 101.982 | 30.151 |
| **37** | 102.882 | 31.977 | **85** | 102.092 | 31.444 | **133** | 100.721 | 31.707 | **181** | 101.996 | 30.066 |
| **38** | 103.095 | 31.035 | **86** | 102.079 | 31.345 | **134** | 100.821 | 31.683 | **182** | 102.001 | 29.957 |
| **39** | 103.033 | 30.939 | **87** | 102.109 | 31.273 | **135** | 100.395 | 31.495 | **183** | 102.071 | 29.909 |
| **40** | 103.345 | 30.989 | **88** | 102.208 | 31.211 | **136** | 100.517 | 31.551 | **184** | 102.063 | 29.827 |
| **41** | 103.211 | 30.911 | **89** | 102.409 | 32.067 | **137** | 100.612 | 31.603 | **185** | 102.121 | 29.711 |
| **42** | 103.111 | 30.886 | **90** | 102.566 | 31.971 | **138** | 100.736 | 31.626 | **186** | 101.788 | 30.328 |
| **43** | 103.035 | 30.844 | **91** | 102.694 | 31.861 | **139** | 100.895 | 31.609 | **187** | 101.868 | 30.189 |
| **44** | 103.121 | 30.841 | **92** | 102.391 | 31.981 | **140** | 100.939 | 31.479 | **188** | 101.876 | 29.976 |
| **45** | 102.202 | 32.207 | **93** | 102.453 | 31.911 | **141** | 100.951 | 31.375 | **189** | 101.877 | 29.822 |
| **46** | 102.394 | 32.048 | **94** | 102.498 | 31.852 | **142** | 100.931 | 31.329 | **190** | 101.678 | 30.275 |
| **47** | 102.566 | 31.962 | **95** | 102.523 | 31.753 | **143** | 100.819 | 31.497 | **191** | 101.746 | 30.172 |
| **48** | 102.701 | 31.865 | **96** | 102.065 | 32.059 | **144** | 100.863 | 31.381 |  |  |  |

**TABLE S2.**Sixteen environmental variables included in the modeling

| **Environment Variable** | **Abbreviation** | **Unit** | **Contribution Rate (%)** |
| --- | --- | --- | --- |
| Maximum temperature of warmest month | bio5 | °C | 8.4 |
| Minimum temperature of coldest month | bio6 | °C | 4.9 |
| Elevation | elev | m | 6.62 |
| Human Footprint | footprint | gha | 0.33 |
| Land cover | landcover | / | 0.13 |
| Seasonal dry matter production | dmps | g/m²/season | 0.01 |
| Gross primary productivity | gpp | g C/m²/year | 1.46 |
| Annual mean temperature | bio1 | °C | 3.48 |
| Annual precipitation | bio12 | mm | 10.44 |
| Temperature seasonality (coefficient of variation) | bio4 | C of V | 9.21 |
| Annual temperature range | bio7 | °C | 50.63 |
| Precipitation of driest month | bio14 | mm | 0.02 |
| Precipitation seasonality (coefficient of variation) | bio15 | C of V | 1.68 |
| Gravel content | t_gravel | - | 1.52 |
| Soil organic carbon content | t_oc | % | 1.16 |
| Soil pH (water) | t_ph_h2o | / | 0.01 |

**TABLE S3.**7 types of models used for modeling the relationship between productivity and suitability

| **Model code** | **Model type** |
| --- | --- |
| a | y=a*exp(b*x) |
| b | y=a*exp(b*x)+c |
| c | y = a*x + b |
| d | y=a*x^^^2+b*x+c |
| e | y=a*ln(x)+b |
| f | y=a*x^^^b |
| g | y=a*x^^^b+c |

**TABLE S4.** Standardized results of various indicators for *P. asiatica*

|  |  |  | **Conventional nutritional components** | | | | | | **Active substance components** | | | **Amino acid components** | | | | | | | | | | | | | | | | |
| --- | --- | --- | --- | --- | --- | --- | --- | --- | --- | --- | --- | --- | --- | --- | --- | --- | --- | --- | --- | --- | --- | --- | --- | --- | --- | --- | --- | --- |
| **Longitude** | **Latitude** | **Suitability** | CFT | CFR | CAH | CPN | DFR | STH | SAN | FLD | TAN | ASP | THR | SER | GLU | GLY | ALA | PRO | CYS | VAL | MET | ILE | LEU | TYR | PHE | HIS | LYS | ARG |
| 102.174 | 31.912 | 0.75 | 4.27 | 4.52 | 0.96 | 7.37 | 4.91 | 15.00 | 6.50 | 9.96 | 3.75 | 0.50 | 0.81 | 0.35 | 4.00 | 0.23 | 0.39 | 1.09 | 0.00 | 1.50 | 0.75 | 1.32 | 0.01 | 1.12 | 1.36 | 1.29 | 1.13 | 2.00 |
| 102.804 | 30.405 | 0.74 | 4.88 | 4.17 | 0.99 | 12.00 | 4.43 | 10.44 | 7.50 | 6.79 | 5.00 | 0.39 | 1.50 | 0.50 | 3.23 | 0.29 | 0.32 | 1.39 | 0.00 | 0.76 | 1.33 | 1.48 | 0.17 | 1.41 | 1.49 | 1.39 | 1.43 | 0.70 |
| 102.235 | 29.926 | 0.72 | 5.19 | 4.52 | 0.96 | 7.80 | 5.64 | 12.34 | 6.50 | 7.92 | 3.00 | 0.43 | 1.17 | 0.42 | 3.47 | 0.33 | 0.39 | 1.57 | 0.00 | 1.16 | 1.17 | 1.15 | 0.63 | 1.31 | 1.32 | 1.29 | 1.28 | 1.23 |
| 102.593 | 30.668 | 0.69 | 5.04 | 4.00 | 0.99 | 10.96 | 4.71 | 11.49 | 7.50 | 6.57 | 0.20 | 0.37 | 1.45 | 0.45 | 3.21 | 0.30 | 0.32 | 1.40 | 0.00 | 0.80 | 1.38 | 1.50 | 0.13 | 1.43 | 1.50 | 1.35 | 1.43 | 0.59 |
| 100.878 | 31.192 | 0.69 | 4.62 | 4.37 | 0.98 | 9.00 | 4.19 | 9.49 | 7.00 | 7.25 | 3.75 | 0.44 | 1.33 | 0.47 | 3.31 | 0.31 | 0.34 | 1.48 | 0.00 | 0.86 | 1.50 | 1.27 | 0.09 | 1.50 | 1.40 | 1.50 | 1.50 | 0.79 |
| 102.676 | 30.973 | 0.68 | 4.42 | 4.42 | 0.95 | 4.80 | 5.06 | 14.24 | 4.50 | 9.51 | 2.50 | 0.49 | 0.75 | 0.33 | 3.96 | 0.25 | 0.42 | 1.04 | 0.00 | 1.46 | 0.67 | 1.35 | 0.01 | 1.03 | 1.40 | 1.18 | 1.05 | 1.94 |
| 102.571 | 30.909 | 0.67 | 5.38 | 4.27 | 0.97 | 6.00 | 6.60 | 11.87 | 5.50 | 6.11 | 2.50 | 0.41 | 1.25 | 0.42 | 3.39 | 0.38 | 0.37 | 1.65 | 0.00 | 1.11 | 1.17 | 1.07 | 0.55 | 1.31 | 1.32 | 1.18 | 1.35 | 1.23 |
| 101.996 | 30.066 | 0.66 | 4.81 | 4.12 | 0.98 | 9.60 | 5.15 | 12.82 | 5.00 | 4.98 | 1.25 | 0.36 | 1.17 | 0.39 | 3.15 | 0.35 | 0.42 | 1.57 | 0.00 | 1.21 | 1.00 | 0.98 | 0.47 | 1.22 | 1.23 | 1.07 | 1.28 | 1.41 |
| 103.111 | 30.886 | 0.65 | 6.04 | 4.23 | 1.00 | 5.45 | 7.61 | 13.81 | 4.50 | 4.75 | 0.16 | 0.38 | 1.08 | 0.39 | 3.56 | 0.33 | 0.39 | 1.48 | 0.00 | 1.16 | 1.00 | 0.94 | 0.53 | 1.22 | 1.23 | 1.07 | 1.28 | 1.32 |
| 102.523 | 31.753 | 0.61 | 6.35 | 4.93 | 0.98 | 9.60 | 3.37 | 9.97 | 2.50 | 12.00 | 3.50 | 0.14 | 0.33 | 0.11 | 1.37 | 0.13 | 0.11 | 0.78 | 0.00 | 0.50 | 0.50 | 0.33 | 0.32 | 0.75 | 0.70 | 0.64 | 0.75 | 0.62 |
| 100.761 | 31.273 | 0.59 | 7.00 | 0.96 | 0.93 | 7.87 | 10.00 | 7.03 | 7.00 | 7.47 | 3.75 | 0.11 | 0.42 | 0.14 | 1.21 | 0.15 | 0.08 | 0.52 | 0.00 | 0.45 | 0.67 | 0.29 | 0.26 | 0.66 | 0.61 | 0.75 | 0.60 | 0.53 |
| 101.081 | 31.026 | 0.59 | 5.58 | 4.77 | 0.94 | 2.40 | 3.22 | 7.12 | 6.00 | 8.38 | 4.25 | 0.31 | 0.92 | 0.33 | 3.39 | 0.46 | 0.45 | 1.83 | 0.00 | 1.11 | 0.83 | 0.04 | 1.39 | 1.03 | 0.96 | 0.64 | 1.35 | 1.32 |
| 100.791 | 31.181 | 0.59 | 4.42 | 2.76 | 1.00 | 9.60 | 5.15 | 7.12 | 6.00 | 9.51 | 4.25 | 0.12 | 0.42 | 0.14 | 1.21 | 0.17 | 0.13 | 0.70 | 0.00 | 0.55 | 1.00 | 0.37 | 0.27 | 0.94 | 0.79 | 0.96 | 0.68 | 0.70 |
| 103.035 | 30.844 | 0.56 | 5.88 | 5.00 | 0.93 | 1.20 | 1.85 | 8.21 | 4.00 | 8.15 | 4.50 | 0.28 | 0.99 | 0.36 | 3.46 | 0.47 | 0.48 | 1.95 | 0.00 | 1.18 | 0.88 | 0.03 | 1.47 | 1.11 | 0.83 | 0.58 | 1.28 | 1.37 |
| 103.095 | 31.035 | 0.55 | 5.96 | 4.87 | 0.92 | 0.96 | 2.26 | 6.17 | 3.50 | 8.38 | 4.75 | 0.26 | 1.08 | 0.39 | 3.56 | 0.50 | 0.50 | 2.00 | 0.00 | 1.21 | 0.67 | 0.08 | 1.50 | 1.13 | 0.79 | 0.75 | 1.28 | 1.50 |
| 103.767 | 32.012 | 0.53 | 3.65 | 1.76 | 0.97 | 8.40 | 5.64 | 8.07 | 5.50 | 6.57 | 3.00 | 0.18 | 0.50 | 0.17 | 1.45 | 0.19 | 0.16 | 0.70 | 0.00 | 0.60 | 0.83 | 0.45 | 0.29 | 0.84 | 0.88 | 0.86 | 0.90 | 0.88 |
| 101.122 | 30.838 | 0.53 | 3.27 | 3.26 | 0.98 | 7.20 | 3.71 | 9.49 | 5.00 | 8.38 | 3.75 | 0.15 | 0.42 | 0.14 | 1.29 | 0.15 | 0.08 | 0.61 | 0.00 | 0.45 | 0.67 | 0.29 | 0.27 | 0.66 | 0.61 | 0.75 | 0.60 | 0.53 |
| 102.329 | 31.857 | 0.49 | 4.04 | 2.26 | 0.95 | 6.00 | 6.60 | 7.12 | 4.50 | 6.11 | 2.50 | 0.16 | 0.50 | 0.17 | 1.37 | 0.17 | 0.11 | 0.70 | 0.00 | 0.50 | 0.83 | 0.33 | 0.29 | 0.75 | 0.70 | 0.86 | 0.75 | 0.70 |
| 102.356 | 31.849 | 0.44 | 1.81 | 3.99 | 0.97 | 9.05 | 3.40 | 5.46 | 8.00 | 0.36 | 1.00 | 0.17 | 0.50 | 0.19 | 1.54 | 0.17 | 0.13 | 0.70 | 0.00 | 0.55 | 1.00 | 0.37 | 0.27 | 0.94 | 0.79 | 0.96 | 0.68 | 0.70 |
| 102.701 | 31.865 | 0.43 | 2.15 | 0.11 | 0.94 | 3.26 | 4.91 | 11.77 | 3.50 | 4.30 | 1.50 | 0.20 | 0.58 | 0.17 | 1.70 | 0.19 | 0.16 | 0.61 | 0.00 | 0.60 | 0.83 | 0.45 | 0.30 | 0.84 | 0.88 | 0.86 | 0.90 | 0.88 |
| 101.429 | 30.462 | 0.42 | 5.19 | 4.27 | 0.90 | 4.80 | 2.74 | 2.37 | 3.00 | 7.25 | 3.25 | 0.13 | 0.33 | 0.11 | 1.13 | 0.13 | 0.11 | 0.78 | 0.00 | 0.50 | 0.50 | 0.33 | 0.30 | 0.75 | 0.70 | 0.64 | 0.75 | 0.62 |
| 102.498 | 31.852 | 0.38 | 2.88 | 1.26 | 0.94 | 3.60 | 4.19 | 4.75 | 4.00 | 4.98 | 1.25 | 0.21 | 0.58 | 0.19 | 1.54 | 0.21 | 0.18 | 0.78 | 0.00 | 0.55 | 1.00 | 0.41 | 0.32 | 0.84 | 0.79 | 0.96 | 0.83 | 0.79 |
| 102.079 | 31.345 | 0.34 | 2.50 | 0.75 | 0.93 | 1.20 | 3.22 | 6.17 | 3.50 | 3.85 | 0.75 | 0.19 | 0.67 | 0.22 | 1.62 | 0.23 | 0.21 | 0.87 | 0.00 | 0.65 | 1.17 | 0.49 | 0.34 | 0.94 | 0.96 | 1.07 | 0.98 | 0.97 |
| 102.109 | 31.273 | 0.33 | 5.58 | 3.77 | 0.88 | 0.96 | 2.26 | 1.90 | 2.00 | 5.66 | 4.00 | 0.10 | 0.25 | 0.08 | 1.05 | 0.10 | 0.05 | 0.87 | 0.00 | 0.40 | 0.33 | 0.25 | 0.24 | 0.56 | 0.53 | 0.54 | 0.53 | 0.44 |
| 101.163 | 31.111 | 0.26 | 1.15 | 0.09 | 0.01 | 0.96 | 2.26 | 7.12 | 5.50 | 4.98 | 2.50 | 0.01 | 0.07 | 0.02 | 0.06 | 0.02 | 0.02 | 0.07 | 0.00 | 0.04 | 0.13 | 0.03 | 0.01 | 0.08 | 0.07 | 0.17 | 0.06 | 0.07 |
| 102.204 | 32.008 | 0.25 | 2.50 | 0.24 | 0.07 | 3.35 | 1.91 | 6.27 | 3.50 | 4.08 | 1.25 | 0.03 | 0.17 | 0.02 | 0.32 | 0.04 | 0.05 | 0.09 | 0.00 | 0.05 | 0.17 | 0.08 | 0.09 | 0.19 | 0.18 | 0.21 | 0.15 | 0.18 |
| 101.406 | 30.865 | 0.23 | 0.15 | 0.65 | 0.03 | 1.80 | 1.05 | 2.61 | 4.50 | 3.62 | 2.25 | 0.09 | 0.50 | 0.17 | 0.81 | 0.13 | 0.16 | 0.52 | 0.00 | 0.30 | 0.83 | 0.25 | 0.18 | 0.56 | 0.53 | 0.75 | 0.45 | 0.53 |
| 102.754 | 30.287 | 0.23 | 0.54 | 0.40 | 0.05 | 2.59 | 0.70 | 3.42 | 5.00 | 3.40 | 2.00 | 0.08 | 0.33 | 0.06 | 0.73 | 0.08 | 0.11 | 0.35 | 0.00 | 0.20 | 0.50 | 0.16 | 0.14 | 0.28 | 0.35 | 0.54 | 0.30 | 0.35 |
| 102.409 | 32.067 | 0.23 | 0.96 | 0.25 | 0.12 | 3.00 | 1.29 | 5.22 | 2.50 | 3.85 | 1.50 | 0.02 | 0.25 | 0.08 | 0.08 | 0.08 | 0.11 | 0.26 | 0.00 | 0.20 | 0.50 | 0.16 | 0.15 | 0.47 | 0.35 | 0.54 | 0.30 | 0.35 |
| 100.633 | 31.717 | 0.21 | 0.65 | 0.85 | 0.07 | 3.84 | 0.32 | 4.03 | 3.00 | 2.94 | 1.25 | 0.05 | 0.33 | 0.11 | 0.48 | 0.06 | 0.08 | 0.26 | 0.00 | 0.15 | 0.50 | 0.12 | 0.11 | 0.28 | 0.26 | 0.43 | 0.23 | 0.26 |
| 101.918 | 30.286 | 0.20 | 0.19 | 0.50 | 0.01 | 1.20 | 0.81 | 2.85 | 4.00 | 3.17 | 1.75 | 0.06 | 0.42 | 0.11 | 0.57 | 0.10 | 0.13 | 0.43 | 0.00 | 0.25 | 0.67 | 0.20 | 0.17 | 0.38 | 0.44 | 0.64 | 0.38 | 0.44 |
| 103.431 | 32.428 | 0.20 | 1.15 | 0.60 | 0.35 | 3.63 | 0.87 | 4.80 | 2.78 | 2.54 | 1.23 | 0.01 | 0.08 | 0.03 | 0.24 | 0.02 | 0.03 | 0.17 | 0.00 | 0.10 | 0.33 | 0.04 | 0.08 | 0.09 | 0.09 | 0.32 | 0.08 | 0.09 |
| 102.209 | 31.859 | 0.19 | 0.77 | 0.75 | 0.14 | 4.20 | 0.57 | 3.80 | 2.00 | 2.72 | 1.00 | 0.04 | 0.17 | 0.06 | 0.40 | 0.06 | 0.05 | 0.17 | 0.00 | 0.15 | 0.33 | 0.12 | 0.12 | 0.28 | 0.26 | 0.43 | 0.23 | 0.26 |
| 102.134 | 31.821 | 0.17 | 0.46 | 1.17 | 0.07 | 4.94 | 0.07 | 4.70 | 0.40 | 0.36 | 0.50 | 0.05 | 0.25 | 0.08 | 0.48 | 0.06 | 0.08 | 0.26 | 0.00 | 0.15 | 0.67 | 0.12 | 0.11 | 0.38 | 0.26 | 0.43 | 0.23 | 0.26 |
| 102.001 | 29.957 | 0.16 | 0.38 | 0.90 | 0.10 | 5.40 | 0.42 | 3.56 | 1.50 | 1.13 | 0.25 | 0.02 | 0.17 | 0.06 | 0.24 | 0.04 | 0.05 | 0.17 | 0.00 | 0.10 | 0.33 | 0.08 | 0.08 | 0.09 | 0.09 | 0.21 | 0.15 | 0.18 |
| 102.374 | 31.976 | 0.15 | 0.58 | 1.00 | 0.09 | 4.80 | 0.08 | 4.27 | 0.50 | 0.45 | 0.75 | 0.03 | 0.08 | 0.03 | 0.16 | 0.04 | 0.03 | 0.09 | 0.00 | 0.10 | 0.50 | 0.08 | 0.06 | 0.19 | 0.18 | 0.32 | 0.15 | 0.18 |

**TABLE S5.** Potential suitable area for *P. asiatica* in different periods(×10^4^ km^2^)

| **Period** | **Climate scenario** | **Low-suitability zone area** | **Moderate-suitability zone area** | **High-suitability zone area** | **Total suitable area** |
| --- | --- | --- | --- | --- | --- |
| Current | / | 5.09 | 1.96 | 0.47 | 7.52 |
| 2050 | SSP126 | 3.46 | 3.17 | 2.49 | 9.12 |
| 2050 | SSP245 | 3.24 | 3.20 | 2.84 | 9.28 |
| 2050 | SSP585 | 3.37 | 3.15 | 3.77 | 10.29 |
| 2090 | SSP126 | 2.82 | 3.36 | 3.34 | 9.52 |
| 2090 | SSP245 | 3.17 | 2.91 | 4.93 | 11.01 |
| 2090 | SSP585 | 2.90 | 2.29 | 7.83 | 13.02 |

**TABLE S6.** Changes in *P. asiatica* in its suitable habitat under climate change scenarios(×10^4^ km^2^)

| **Period** | **Climate scenario** | **Habitat area** | **Loss area** | **Stable area** | **Gain area** |
| --- | --- | --- | --- | --- | --- |
| 2050 | SSP126 | 9.12 | 0.49 | 7.03 | 2.09 |
| 2050 | SSP245 | 9.28 | 0.41 | 7.10 | 2.17 |
| 2050 | SSP585 | 10.29 | 0.38 | 7.14 | 3.15 |
| 2090 | SSP126 | 9.52 | 0.35 | 7.17 | 2.35 |
| 2090 | SSP245 | 11.01 | 0.28 | 7.24 | 3.77 |
| 2090 | SSP585 | 13.02 | 0.20 | 7.32 | 5.71 |

**Table S7.** Potential cultivation area of *P. asiatica* in different periods (×10^4^ km^2^)

| **Period** | **Climate scenario** | **Third-Level Zones**  **Area** | **Second-Level Zones**  **Area** | **First-Level Zones**  **Area** | **Total Cultivation Area** |
| --- | --- | --- | --- | --- | --- |
| Current | / | 5.68 | 0.89 | 0.23 | 6.80 |
| 2050 | SSP126 | 4.78 | 2.21 | 1.71 | 8.70 |
| 2050 | SSP245 | 4.53 | 2.37 | 1.97 | 8.87 |
| 2050 | SSP585 | 4.49 | 2.48 | 2.82 | 9.79 |
| 2090 | SSP126 | 4.21 | 2.63 | 2.31 | 9.15 |
| 2090 | SSP245 | 4.13 | 2.53 | 3.88 | 10.54 |
| 2090 | SSP585 | 3.63 | 1.95 | 7.00 | 12.58 |
